# The Amadori rearrangement as glycoconjugation method: Synthesis of non-natural *C*-glycosyl type glycoconjugates

**DOI:** 10.3762/bjoc.8.185

**Published:** 2012-09-25

**Authors:** Katharina Gallas, Gerit Pototschnig, Florian Adanitsch, Arnold E Stütz, Tanja M Wrodnigg

**Affiliations:** 1 Glycogroup, Institute of Organic Chemistry, Technical University Graz, Stremayrgasse 9, A-8010 Graz, Austria

**Keywords:** Amadori rearrangement, *C*-glycosyl type glycoconjugates, carbohydrate elongation, non-natural carbohydrate conjugates

## Abstract

The Amadori rearrangement was investigated as a potential method for the conjugation of carbohydrate moieties to suitable amino components. Starting from selected aldoheptoses, which are readily available by means of the Kiliani–Fischer C-elongation reaction of the corresponding aldohexoses, glycoconjugates presenting D-*gluco*, D-*manno* and D-*galacto* as well as GlcNAc motifs have been synthesised. Following this strategy, non-natural *C*-glycosyl type glycoconjugates, which can be utilised as building blocks for the composition of larger molecular constructions, are available by a very short synthetic approach.

## Introduction

**Scheme 1:**
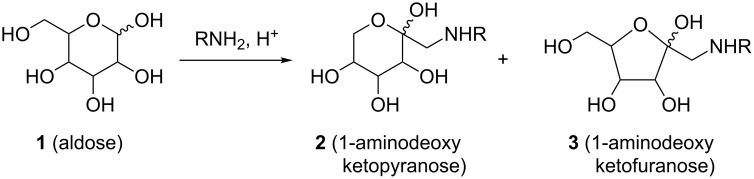
Amadori rearrangement.

Glycoconjugates such as glycoproteins, glycopeptides, glycolipids and peptidoglycans are ubiquitous in nature [1]. They are found on cell surfaces and are responsible for processes such as cell–cell interaction, recognition and communication. In order to investigate and elucidate their many functions, reliable synthetic methods for the conjugation of carbohydrates to biomolecules are of crucial importance [2]. Several ligation strategies are described in the literature, such as chemical ligation [3], conjugation by means of click chemistry (Huisgen cycloaddition) [4–5], glycosylation protocols [6–7], and Staudinger ligation [8], just to mention the most prominent examples. However, many applications in this respect require a ligation method that is functional in aqueous medium, and, for economic purposes in general, protecting-group manipulations should be kept to a minimum. Considering these aspects, we were interested in probing the Amadori rearrangement as a conjugation method for the synthesis of glycoconjugates. The Amadori rearrangement (AR) allows the formation of 1-aminodeoxyketoses **2** and **3** from the respective aldose **1** and a suitable amine under acid catalysis (Scheme 1).

The reaction is known to be very sensitive to the nature of the carbohydrate substrate, the acid catalyst, and the amino component, as well as to the temperature and duration. Additionally, some challenges accompany the reaction limiting its synthetic use, several steps during the introduction of the amine and the subsequent isomerisation to the ketose are reversible, and furthermore, the product itself can enter the Maillard reaction cascade [9–10] pathway. Consequently, a range of side and degradation products can be formed. In general, the rearrangement product can occur as a mixture of different isomers, and in principal the furanoid as well as pyranoid in their α- and β-anomeric forms can be obtained. Thus, isolation of the rearrangement product can be challenging and tedious, and only a few preparatively useful examples of the Amadori rearrangement are known in the literature [11–20]. However, this reaction accomplishes the introduction of various amines onto the position C-1 of aldoses and at the same time the isomerisation to the corresponding 1-aminodeoxyketose without the requirement for protecting-group manipulations, and is thus a very valuable reaction sequence [21–24]. We have succeeded in optimising the reaction conditions for the Amadori rearrangement when introducing D-*glycero*-D-*gulo*-aldoheptose as the starting material. In this special case, all substituents of the resulting 1-aminodeoxy ketose adopt an equatorial position in the ^5^*C*_2_ pyranoid conformation, which is a strong driving force providing excellent preparative yields [25]. In general, this reaction leads to non-natural *C*-glycosyl type glycoconjugates, which are of particular interest for biological investigation because of their chemical stability towards physiological hydrolysis of the glycosidic linkage, compared to the naturally occurring *O*- as well as *N*-glycosides [26].

A broad application of this reaction is not feasible as yet. However, according to its mechanism, the scope of the Amadori rearrangement could well comprise a variety of useful conjugation reactions, including mono- as well as oligo- and polyvalent amines, and the carbohydrate decoration of functionalized surfaces. Fields of application concern the construction of oligovalent glycoconjugates, such as glycoclusters and glycodendrimers, the modification of surfaces (glycosylated surfaces, e.g., “glyco-chips”, gold, polystyrene plates, glass, nanoparticles), or labelling and imaging of carbohydrates (fluorescence markers, biotin, photolabelling) [27–28].

Herein we describe the application of the Amadori rearrangement as a key step for the synthesis of non-natural *C*-glycosyl type glycoconjugates in the D-*manno*, D-*galacto* as well as GlcNAc series starting from the respective aldoheptoses and suitable amino components. Aldoheptoses were synthesised by C-elongation of the corresponding hexoses and subsequent reductive hydrolysis following the Kiliani–Fischer cyanohydrin reaction procedure.

## Results and Discussion

### C-Elongation

**Scheme 2:**
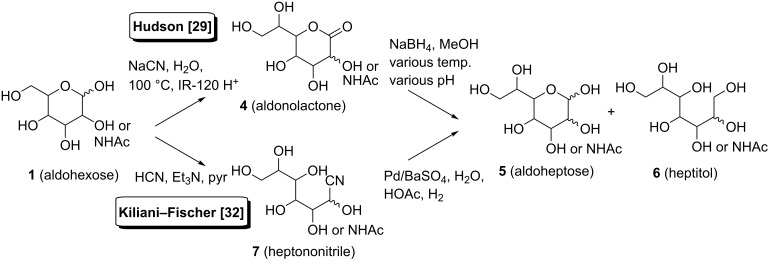
C-Elongation using the sodium cyanide/sodium borohydride and HCN/Pd(BaSO_4_) method.

In an initial C-elongation attempt, with the aim to avoid HCN as C-synthon, we decided to follow a protocol employing sodium cyanide, as described by Hudson [29] (Scheme 2). By this reaction sequence, starting from aldohexose **1**, the obtained heptonolactones **4** are expected to be formed as a mixture of diastereomers at position C-2. Consequently, the respective aldoheptoses **5**, obtained by reduction of the lactones employing sodium borohydride, will be present as C-2 diastereomers as well. However, during the Amadori rearrangement this centre will be oxidised to a keto group, thus the stereochemical outcome of the chain elongation is not of relevance and therefore a separation is not necessary. As a side product, the fully reduced heptitol **6** is expected to be formed.

**Scheme 3:**
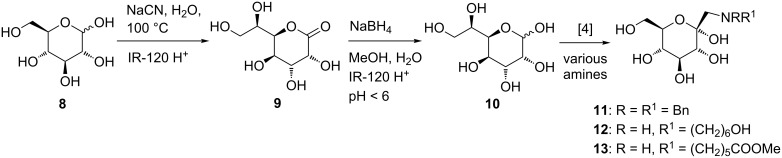
C-Elongation as well as Amadori rearrangement in the D-*gluco* series.

To probe this reaction sequence, i.e., C-elongation followed by Amadori rearrangement, D-glucose (**8**) was employed as a model substrate, since in this case NMR data of the obtained D-*glycero*-D-*gulo* aldoheptose **10** can be compared with a commercially available sample (Scheme 3). An aqueous solution of D-glucose (**8**) was treated with sodium cyanide at 100 °C for two days. Hydrolysis of the resulting cyanohydrin to aldonolactone **9** was accomplished by treatment with strong acidic ion-exchange resin IR-120 H^+^. In contrast to the described reduction employing sodium amalgam, we preferred sodium borohydride, which has been reported as a suitable agent for the reduction of lactones to the corresponding acetals when the pH value is kept below 6 [30]. In the D-*gluco* series, D-*glycero*-D-*gulo*-aldoheptose **10** was isolated in 81% yield, and no C-2 epimer was detected, as shown by comparison of the NMR data with a commercial sample [31]. Aldoheptose **10** was taken to the Amadori rearrangement without further purification. Three typical amines, dibenzylamine, 6-aminohexanol as well as 6-aminohexanoic acid methyl ester hydrochloride were investigated [25]. In all cases the corresponding Amadori products **11**, **12** as well as **13** were formed, which was confirmed by comparison of the NMR data [25].

Despite thorough investigation of the conditions for the reduction with sodium borohydride by adjusting the pH value, the amount of reagent, and the temperature, it was not possible to obtain the desired aldoheptose in the D-*galacto* as well as the D-*manno* series in satisfactory yields (Scheme 2). In all cases, a mixture of the unreacted lactone **4**, the fully reduced alditol **6** as the main product, and a small amount of the desired aldoheptose **5** was formed. When *N*-acetyl-D-glucosamine was subjected to this reduction method, the corresponding heptitol **6** was isolated as the main product under all conditions tried.

**Scheme 4:**
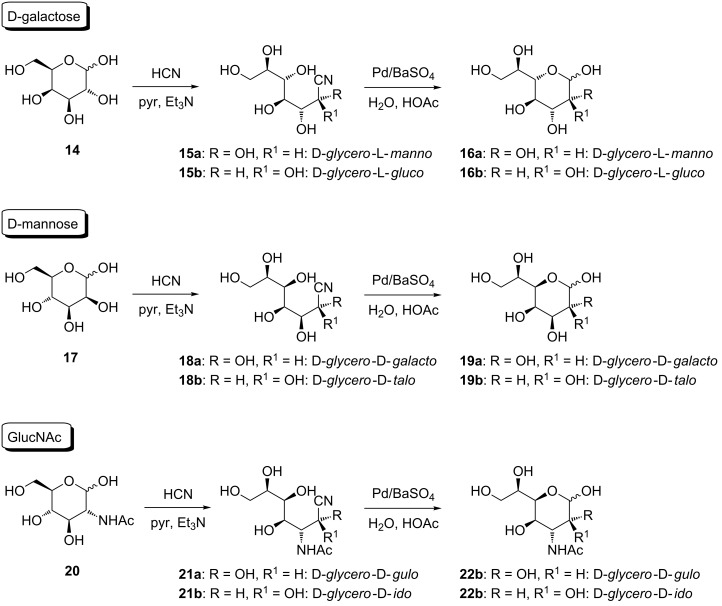
C-Elongation method by modified Kiliani–Fischer protocol from of D-galactose, D-mannose as well as GlcNAc.

These unsatisfactory results caused us to change the strategy for the C-elongation reaction sequence. Following a slightly modified procedure of the Kiliani–Fischer cyanohydrin reaction reported by Kuhn and Baschang [32], aldoses were exposed to hydrocyanic acid in pyridine and triethylamine, replacing water as solvent, and thereby shortening the reaction time from 15 to 3 days (Scheme 2). A small amount of the reaction mixture was per-*O*-acetylated in order to monitor the reaction by TLC, thus avoiding hydrolysis of the cyanohydrin on the TLC plate, and to track the progress of the reaction. The subsequent reductive hydrolysis of the heptononitrile **7** to the desired aldoheptoses **5** was performed as described by these authors employing Pd/BaSO_4_ in water and acetic acid. Thus, the corresponding heptose **5**, in some cases as mixtures of C-2 epimers, were isolated in preparatively useful yields. With this method in hand, D-galactose (**14**), D-mannose (**17**) as well as GlcNAc (**20**) were successfully converted to the corresponding aldoheptoses (Scheme 4), namely D-*glycero*-L-*manno*/L-*gluco*-heptopyranose **16a** and **16b**, D-*glycero*-D-*galacto*/D-*talo*-heptopyranose **19a** and **19b**, and 3-acetamido-3-deoxy-D-*gluco*-D-*ido*/D-*gulo*-heptopyranose **22a** and **22b**, via the respective cyanohydrins **15**, **18** and **21**, in preparatively expedient yields.

### Amadori rearrangement

With these aldoheptoses in hand we investigated the Amadori rearrangement. The product mixture, containing mainly a C-2 epimeric mixture of α,β-aldoheptoses and minor amounts of side products from the reductive hydrolysis, was used without further purification. It was expected that the respective aldoheptose would undergo predominantly the Amadori rearrangement, whereas the remaining aldohexose and side products would be rather unreactive under the reaction conditions employed.

**Scheme 5:**
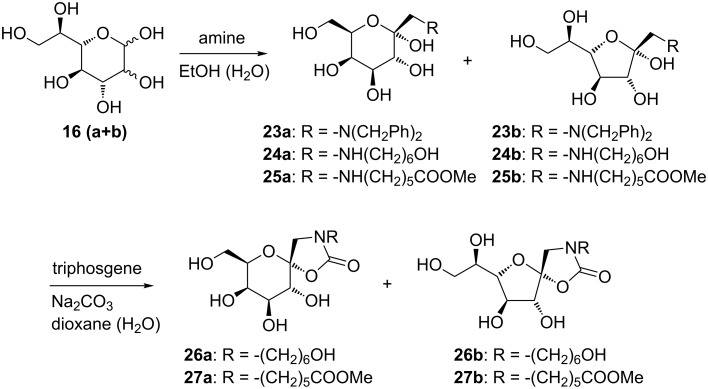
Amadori rearrangement in the D-*galacto* series.

In the D-*galacto* series, the mixture containing D-*glycero*-L-*manno*/L-*gluco*-heptopyranose **16a** and **16b** was treated with dibenzylamine in ethanol and 1,4-dioxane as co-solvent, employing one equivalent of acetic acid, to give, after purification by silica-gel chromatography, predominantly the α-anomer of 1-(*N*,*N*-dibenzyl)amino-1-deoxy-α-D-*galacto*-hept-2-ulose in a yield of 71% and as mixture of the pyranoid **23a** and furanoid **23b** forms in a ratio of 3:1 (Scheme 5). 6-Aminohexanol as amino component in aqueous ethanol, containing one equivalent of acetic acid gave, after three days at 50 °C, 1-(6-hydroxyhexylamino)-1-deoxy-α-D-*galacto*-hept-2-ulose in a yield of 74% and with a 4:1 ratio of the pyranoid **24a** and furanoid **24b** forms. In the case of 6-aminohexanoic acid methyl ester hydrochloride, the Amadori rearrangement was performed in ethanol with 1,4-dioxane and water as co-solvent in the presence of triethylamine in order to liberate the free amine from the corresponding ammonium hydrochloride. After purification, 1-(5-(methoxycarbonyl)pentylamino-1-deoxy-α-D-*galacto*-hept-2-ulose was also obtained as a mixture of pyranoid **25a** and furanoid **25b** forms with a 5:1 ratio and a combined yield of 35%. The Amadori products featuring a secondary amine at position C-1, compounds **24** and **25**, were further reacted with triphosgene [25]. In this reaction, the anomeric hydroxy group and the amine at position C-1 formed a cyclic carbamate, thereby stabilising the hemiacetal at the anomeric position. Compounds **24** (**a** and **b**) gave the corresponding 1-*N*,2-*O*-cyclic carbamates in 95% yield with a 3:2 ratio of the pyranoid **26a** and furanoid **26b** forms, and compounds **25** (**a** and **b**) reacted accordingly and gave cyclic carbamates **27a** and **27b** in a 5:3 ratio and a combined yield of 50%.

**Scheme 6:**
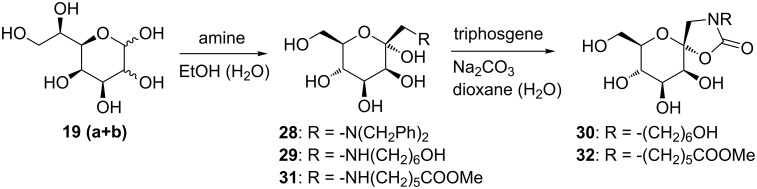
Amadori rearrangement in the D-*manno* series.

In the D-*manno* series, the C-elongation employing HCN in pyridine and triethylamine and subsequent reductive hydrolysis of the corresponding nitriles **18** (**a** and **b**) gave very good conversion to D-*glycero*-D-*galacto* heptose **18a**, together with a minor amount of D-*glycero*-D-*talo*-heptopyranose **19b** (Scheme 4). From the NMR analysis of the crude product mixture it was obvious that the predominant product was the β-anomer of D-*glycero*-D-*galacto*-aldoheptose. This was verified by ^1^H NMR data analysis, the β-anomer presenting a coupling constant of *J*_1,2_ = 7.8 Hz between protons H-1 and H-2. The Amadori rearrangement with dibenzylamine gave 1-(*N*,*N*-dibenzylamino)-1-deoxy-α-D-*manno*-hept-2-ulopyranose (**28**) in 72% yield, exclusively (Scheme 6). Likewise, with 6-aminohexanol as amino component, the Amadori rearrangement of aldoheptoses **19** (**a** and **b**) gave the corresponding 1-(*N*-6-hydroxyhexyl)amino-1-deoxy-α-D-manno-hept-2-ulose (**29**), exclusively in an isolated yield of 70%. No β-anomer formation could be observed from NMR analysis. Treatment of compound **29** with triphosgene and sodium carbonate in 1,4-dioxane and H_2_O gave 1-*N*-(hydroxyhexyl)amino-α-D-*manno*-1-*N*,2-*O*-carbamate **30** in 75% yield. With 6-aminohexanoic acid hydrochloride, the Amadori rearrangement, employing triethylamine for liberation of the free amine from the hydrochloride, gave a 90% yield of the α-anomer **31**. Its treatment with triphosgene provided 1-*N*-2-*O*-cyclic carbamate **32** in 50% yield.

**Scheme 7:**
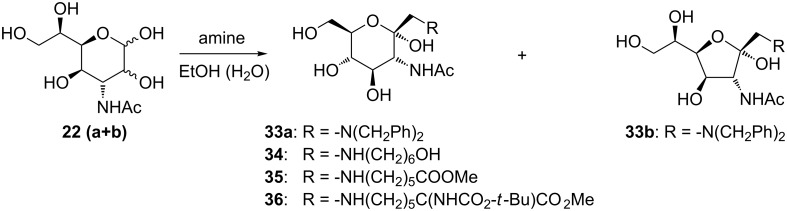
Amadori rearrangement in the GlcNAc series.

3-Acetamido-3-deoxy-D-*gluco*-D-*id*o/D-*gulo*-heptopyranose **22** (**a** and **b**) gave under Amadori rearrangement conditions with dibenzylamine predominantly 1-(*N*,*N*-dibenzyl)amino-3-acetamido-3-deoxy-α-D-*gluco*-heptulose (**33a**, Scheme 7), and a small amount of the corresponding furanoid form **33b** could be detected by NMR analysis. 6-Aminohexanol as amino component gave exclusively the α-anomer of 1-(*N*-6-hydroxyhexyl)amino-3-acetamido-D-*gluco*-heptulose **34** in the pyranoid form and in 50% yield after purification by column chromatography. Methyl 6-aminohexanoate hydrochloride gave the corresponding α-pyranose **35**, albeit in only 25% yield. With the α-*N*-Boc protected L-lysine derivative Boc-L-Lys(Z)-OMe, the Amadori rearrangement gave the 1-[(5*S*-*tert*-butoxylcarbonylamino)-6-methoxycarbonylpentyl]amino-3-acetamido-1,3-dideoxy-α-D-*gluco*-hept-2-ulopyranose (**36**) in the pyranoid form in 30% yield.

## Conclusion

In conclusion, we have successfully developed a protocol for the conjugation of carbohydrate moieties to amine containing molecules through the Amadori rearrangement. By reaction of respective aldoheptoses, non-natural *C*-glycosyl type glycoconjugates presenting the sugar motif in D-*gluco*, D-*manno*, and D-*galacto* as well as GlcNAc configuration are available. This indicates that the Amadori rearrangement is a valuable tool in the repertoire of ligation methods, with the advantage that no protecting-group manipulations are necessary in the carbohydrate moiety and the reaction can be carried out in aqueous environment offering obvious advantages in glycobiology. In preliminary studies, several disaccharidic structures, such as cellobiose, lactose and *N*-DTPM-2-aminodeoxylactose [33], were successfully converted to the respective *C*-glycosyl type glycoconjugates by this method. Optimisation of yields is currently under progress [34].

## Experimental

**General methods:** NMR spectra were recorded on a Bruker Ultrashield spectrometer at 300.36 or 75.53 MHz, in methanol-*d*_4_ or D_2_O as indicated. Chemical shifts are listed in parts per million (ppm) employing residual, nondeuterated solvent as the internal standard. Structures of crucial intermediates were unambiguously assigned by 1D-TOCSY and HSQC experiments. Optical rotations were measured on a Perkin Elmer 341 polarimeter at a wavelength of 589 nm and a path length of 10 cm at 20 °C. Analytical TLC was performed on precoated aluminium plates Silica Gel 60 F_254_ (E. Merck 5554), detected with UV light (254 nm), 10% vanillin/sulfuric acid and ceric ammonium molybdate (100 g ammonium molybdate/8 g ceric sulfate in 1 L 10% H_2_SO_4_), and heated on a hotplate. Preparative TLC was performed on precoated glass plates silica gel 60 F_254_, 0.5 mm (E. Merck 5744). For column chromatography silica gel 60 (230–400 mesh, E. Merck 9385) was used.

**General procedure for generation of HCN** [35]**:** Equipment for all reactions in which HCN or cyanides were involved, was placed in a well-ventilated hood. For safety, an electrochemical sensor for HCN detection was used. The required amount of HCN was freshly prepared by adding a saturated NaCN solution dropwise to aqueous sulfuric acid (60%) at 80 °C. HCN was transferred in a nitrogen stream through a drying column (CaCl_2_) and collected in a cooling trap at −12 °C. Waste solutions containing cyanides were treated with aqueous sodium hypochlorite (10%).

**General procedure for preparation of aldoheptoses** [32]**:** The respective aldose was dissolved in pyridine, then triethylamine (0.08 equiv) and HCN (8 equiv) were added, and the reaction was kept in a sealed flask at room temperature for several days. For monitoring of the reaction by TLC, a small amount of the reaction mixture was subjected to a per-*O*-acetylation. After complete consumption of the starting material, the remaining HCN and solvents were removed under reduced pressure. The remaining material was azeotroped/co-evaporated with EtOH, and subsequently the products were precipitated by treatment of the residue with ether/ethylacetate 1:1 v/v at 4 °C. The obtained heptononitriles were used for the next step immediately. For the reductive hydrolysis, the respective heptononitrile was dissolved in water, and then acetic acid (1.1 equiv) and Pd/BaSO_4_ (0.6 g per g heptononitrile) were added, and the reaction mixture was stirred under a hydrogen atmosphere at ambient pressure and temperature until the starting material was consumed. The catalyst was removed by filtration and the pH adjusted with ion-exchange resin (IR-120 H^+^) to 2. Filtration of the resin and removal of the solvent under reduced pressure afforded a mixture of products, containing mainly the respective aldoheptoses. This mixture was used for the Amadori rearrangement immediately without further purification.

**General method A (Amadori rearrangement with free amines)** [25]**:** The respective aldose was dissolved in abs. EtOH, and then 1.2 equiv of the free amine and 1.2 equiv of AcOH were added, and the reaction mixture was stirred at 40 °C until TLC showed satisfactory conversion of the starting material. The reaction mixture was concentrated under reduced pressure, and the obtained products were separated by flash chromatography with the solvent system indicated in the experimental section.

**General method B (Amadori rearrangement with amine hydrochlorides)** [25]**:** The respective amine hydrochloride was dissolved in abs. EtOH, 1 equiv of Et_3_N was added, and the mixture was stirred for 20 min at rt. To this mixture the aldose was added, and the reaction mixture was stirred at 40 °C until TLC showed satisfactory conversion of the starting material. The reaction mixture was concentrated under reduced pressure and the obtained residue was purified by flash chromatography with the solvent system indicated in the experimental section.

**General method C (cyclic carbamate formation with triphosgene)** [25]**:** The Amadori rearrangement compound was dissolved in water, and in indicated cases 1,4-dioxane was added to enhance the solubility. A large access of Na_2_CO_3_, typically >6 equiv, was added at 0 °C. After 15 min triphosgene was added and the reaction mixture stirred for 20 min at 0 °C followed by stirring at room temperature until TLC showed conversion of the starting material. The reaction was concentrated under reduced pressure and the obtained compound mixture separated by flash chromatography with the solvent mixture indicated in the experimental section.

**1-(*N*,*N*-Dibenzylamino)-1-deoxy-α-D-*galacto*-hept-2-ulopyranose (23a) and -furanose (23b):** The general procedure for the preparation of aldoheptoses was applied to D-galactose (**14**, 3 g, 16.7 mmol) by using pyridine (45 mL), triethylamine (0.18 mL, 1.3 mmol, 0.08 equiv) and HCN (5.6 mL, 0.14 mmol, 8.5 equiv) as described. After five days complete consumption of the aldose was indicated by TLC (per-*O*-acetylation of a sample of the reaction mixture was performed, EE/C (ethyl acetate/cyclohexane) 1:1 v/v) and 5 g of the crude heptononitriles **15a** and **15b** was obtained. This mixture of heptononitriles (2.5 g) was treated in water (50 mL) containing acetic acid (0.75 mL, 1.1 equiv) with Pd/BaSO_4_ (1.5 g) under a hydrogen atmosphere at ambient pressure. After TLC (CHCl_3_/MeOH 1:1 v/v containing 0.25% concd NH_4_OH) indicated complete consumption of the starting material and workup as described, 3 g of crude aldoheptoses **16a** and **16b** were obtained, which were used for the Amadori rearrangement immediately without any further purification. General procedure A was applied to the mixture of aldoheptose **16a** and **16b** (400 mg) employing EtOH (4 mL) and 1,4-dioxane as co-solvent, dibenzylamine (360 µL, 1.9 mmol) and acetic acid (110 µL, 1.9 mmol). The reaction mixture was stirred at 50 °C for five days. Column chromatography (EE/MeOH 15:1 v/v) gave 480 mg of a mixture of pyranoid **23a** and furanoid **23b** product in a ratio of 3:1 and in an overall yield of 70%. α-Anomer: [α]_D_ = +20 (*c* 1.4, MeOH); ^1^H NMR (methanol-*d*_4_) δ 7.37–7.35 (m, 10H, Ph), 4.06 (d, *J* = 13.4 Hz, 2H, CH_2_Ph), 4.06–4.00 (m, 1H, H-6), 3.93 (dd, *J*_4,5_ = 3.4 Hz, *J*_5,6_ = 3.5 Hz, 1H, H-5), 3.78–3.71 (m, 2H, H-4, H-7), 3.69–3.65 (m, 1H, H-7), 3.55 (d, 2H, CH_2_Ph), 3.52 (d, *J*_3,4_ = 9.8 Hz, 1H, H-3), 3.05 (d, *J*_1,1_ = 13.4 Hz, 1H, H-1), 2.72 (d, 1H, H-1); ^13^C NMR δ 139.6, 130.5, 129.8, 129.6 (Ph), 98.7 (C-2), 72.6, 72.4, 71.5, 70.9 (4C, C-3, C-4, C-5, C-6), 62.6 (C-7), 60.0 (2C, CH_2_Ph), 58.8 (C-1); anal. calcd for C_21_H_27_NO_6_: C, 64.77; H, 6.43; found: C, 63.09; H, 6.52.

**1-(*N*-(6-Hydroxyhexyl)amino)-1-deoxy-α-D-*galacto*-hept-2-ulopyranose (24a) and -furanose (24b):** Following general method A, the aldoheptoses **16a** and **16b** (370 mg) were treated with 6-aminohexanol (200 mg, 1.76 mmol, 1 equiv) in EtOH (5 mL) and 1,4 dioxane as co-solvent in the presence of acetic acid (0.1 mL, 1.76 mmol, 1 equiv) at 50 °C for three days. Silica-gel chromatography (CHCl_3_/MeOH 4:1 v/v containing 10% of concd NH_4_OH) gave the respective Amadori compound in a mixture of α-pyranose **24a** and α-furanose **24b** in a ratio of 4:1 and an overall yield of 74%. Pyranoid α-anomer **24a**: ^1^H NMR (methanol-*d*_4_) δ 4.00 (m, 1H, H-6), 3.88 (m, 1H, H-5), 3.81–3.69 (m, 3H, H-4, 2 × H-7), 3.63 (d, *J*_3,4_ = 9.6 Hz, 1H, H-3), 3.57 (t, 2H, H-13), 3.29 (d, *J_1,1_* = 12.5 Hz, 1H, H-1), 3.15 (d, 1H, H-1), 3.02 (t, 2H, H-8), 1.82–1.68 (m, 2H, H-9), 1.64–1.53 (m, 2H, H-12), 1.51–1.40 (m, 4H, H-10, H-11); ^13^C NMR δ 96.7 (C-2), 73.6 (C-6), 71.8, 71.5, 71.1 (3C, C-3, C-4, C-5), 62.9 (C-7), 62.7 (C.13), 54.6 (C-1), 49.7 (C-8), 33.3 (C-12), 27.5, 27.1, 26.5 (3C, C-9, C-10, C-11); anal. calcd for C_13_H_27_NO_7_: C, 50.50; H, 8.82; found: C, 50.46; H, 8.88.

**1-(*N*-(5-(Methoxycarbonyl)pentyl)amino)-1-deoxy-α-D-*galacto*-hept-2-ulopyranose (25a) and -furanose (25b):** Following general method B, a solution of 6-aminohexanoic acid methyl ester hydrochloride (480 mg, 2.7 mmol, 1.0 equiv) in EtOH (6 mL) with 1,4-dioxane (1 mL) as co-solvent containing Et_3_N (370 μL, 2.6 mmol, 1.0 equiv) was applied to the aldoses **16a** and **16b** (560 mg, 2.7 mmol). The reaction mixture was stirred at 60 °C for one day. The solvents were removed under reduced pressure and silica-gel chromatography (CHCl_3_/MeOH 4:1 v/v containing 10% of concd NH_4_OH) gave 300 mg of a mixture of pyranose **25a** and furanose **24b** in 5:1 ratio and 35% yield as a yellow oil. Pyranose **25a** signals: ^1^H NMR (methanol-*d*_4_) δ 3.91–3.84 (m, 1H, H-6), 3.81–3.73 (m, 1H, H-5), 3.68 (dd, *J*_4,5_ = 3.2 Hz, *J*_3,4_ = 9.7 Hz, 1H, H-4), 3.63–3.57 (m, 2H, H-7), 3.56 (s, 3H, OCH_3_), 3.53 (d, 1H, H-3), 2.79 (d, *J*_1,1_ = 12.1 Hz, 1H, H-1), 2.73 (d, 1H, H-1), 2.62–2.48 (m, 2H, H-8), 2.25 (t, 2H, H-12), 1.60–1.48 (m, 2H, H-10), 1.48–1.40 (m, 2H, H-11), 1.33–1.23 (m, 2H, H-9); ^13^C NMR δ 175.9 (C-13), 98.2 (C-2), 72.8, 72.4, 71.6, 71.3 (3C, C-3, C-4, C-5, C-6), 62.9 (C-7), 56.0 (C-1), 52.1 (OCH_3_), 50.8 (C-8), 34.7 (C-12), 30.0 (C-11), 27.7 (C-9), 25.9 (C-10); anal. calcd for C_14_H_27_NO_8_: C, 49.87; H, 8.09; found: C, 49.79; H, 8.11.

**1-(*N*-(6-Hydroxyhexyl)amino)-1-*N*,2-*O*-carbonyl-1-deoxy-β-D-galacto-hept-2-ulopyranose 26a and -furanose 26b:** General method C was applied to compounds **24a** and **24b** (349 mg) in H_2_O (10 mL) and 1,4-dioxane (2 mL), employing Na_2_CO_3_ (1.3 g, 11.8 mmol, 6 equiv) and triphosgene (0.6 g, 2.0 mmol, 1.8 equiv). Silica-gel chromatography (CHCl_3_/MeOH 3:1 v/v containing 1% of concd NH_4_OH) gave a mixture of pyranose **26a** and furanose **26b** (350 mg, 95%) in a 3:2 ratio. Pyranose **26a** signals: ^1^H NMR (methanol-*d*_4_) δ 4.07–3.99 (m, 2H, H-5, H-6), 3.87 (d, *J*_1,1_ = 10.0 Hz, 1H, H-1), 3.84–3.79 (m, 2H, H-3, H-4), 3.79–3.69 (m, 2H, H-7), 3.58 (t, 2H, H-13), 3.47 (d, 1H, H-1), 3.32–3.25 (m, 2H, H-8), 1.67–1.49 (m, 4H, H-9, H-12), 1.49–1.30 (m, 4H, H-10, H-11); ^13^C NMR δ 158.8 (C=O), 104.6 (C-2), 75.7 (C-5), 71.9, 70.6, 70.8 (3C, C-3, C-4, C-6), 62.9 (C-13), 62.4 (C-7), 53.5 (C-1), 44.5 (C-8), 33.5 (C-12), 28.1, 27.4, 26.5 (3C, C-9, C-10, C-11); anal. calcd for C_14_H_25_NO_8_: C, 50.17; H, 7.53; found: C, 50.10; H, 7.59.

**1-(*N*-(5-Methoxycarbonylpentyl)amino)-1-*N*,2-*O*-carbonyl-1-deoxy-β-D-*galacto*-hept-2-ulopyranose (27a) and -furanose (27b):** General method C was applied to the mixture of Amadori products **26a** and **26b** (200 mg, 0.6 mmol), Na_2_CO_3_ (680.0 mg, 6.4 mmol, 6.0 equiv) and triphosgene (320 mg, 1.1 mmol, 1.8 equiv) in H_2_O (7 mL) and 1,4-dioxane as co-solvent (1 mL). When TLC indicated complete consumption of the starting material, the solvents were removed under reduced pressure, and silica-gel chromatography (CHCl_3_/MeOH 6:1 v/v containing 1% of concd NH_4_OH) gave a mixture of cyclic carbamates in the pyranoid **27a** and furanoid **27b** form in a ratio of 5:3 (280 mg, 50%) as a yellow oil. Pyranose **27a** signals: ^1^H NMR (methanol-*d*_4_) δ 4.06–3.94 (m, 2H, H-5, H-6), 3.87–3.79 (m, 2H, H-1, H-4), 3.87–3.70 (m, 3H, H-3, H-7), 3.68 (s, 3H, OCH_3_), 3.45 (d, *J*_1,1_ = 9.8 Hz, 1H, H-1), 2.37 (t, 2H, H-12), 1.72–1.55 (m, 4H, H-9, H-11), 1.42–1.31 (m, 3H, H-10); ^13^C NMR δ 176.0 (C-13), 158.6 (NCO), 104.5 (C-2), 75.7, 72.0, 70.9, 70.8 (4C, C-3, C-4, C-5, C-6), 62.5 (C-7), 53.4 (C-1), 52.1 (OCH_3_), 44.3 (C-8), 34.7 (C-12), 27.8 (C-9), 27.0 (C-10), 25.7 (C-11); anal. calcd for C_15_H_25_NO_9_: C, 49.61; H, 6.95; found: C, 49.58; H, 6.97.

**1-(*N*,*N*-Dibenzylamino)-1-deoxy-α-D-*manno*-hept-2-ulopyranose (28):** General method for sugar elongation was applied to D-mannose **17** (3 g, 16.7 mmol), pyridine (50 mL), triethylamine (0.18 mL, 1.3 mmol, 0.08 equiv) and HCN (4.5 mL, 0.11 mmol, 6.9 equiv). After five days, complete consumption of the aldose was indicated by TLC, and 8 g of the crude heptononitrile **18** (**a** and **b**) were obtained. For the reductive hydrolysis the heptononitriles **18** (4.5 g) were treated as described employing dist. water (90 mL), acetic acid (1.35 mL, 1.1 equiv) and Pd/BaSO_4_ (2.7 g). After TLC (CHCl_3_/MeOH 1:1 v/v containing 0.25% concd NH_4_OH) showed complete consumption of the starting material, and workup with ion-exchange resin IR-120 H^+^, 3.7 g of crude aldoheptoses **19a** and **19b** were obtained. NMR analysis of the crude product mixture indicated the D-*glycero*-D-*galacto*-β-aldoheptose **19a** as the predominant compound (4.48 ppm, *J*_1,2_ = 7.8 Hz, 1H, H-1β) next to its α-anomer (5.14 ppm, *J*_1,2_ = 3.6 Hz, 1H, H-1α) in a ratio of 5:2. This compound mixture was used for the Amadori rearrangement. General procedure A was applied to the mixture of aldoheptoses **19** (400 mg) employing EtOH (5 mL) and 1,4-dioxane (1 mL) as cosovlent, dibenzylamine (360 µL, 1.9 mmol) and acetic acid (110 µL, 1.9 mmol). The reaction mixture was stirred at 50 °C for three days. Column chromatography (EE/MeOH 20:1 v/v) gave product **28** in a yield of 72% (540 mg). α-Anomer **28**: [α]_D_ = +20 (*c* 1.1, MeOH); ^1^H NMR (methanol-*d*_4_) δ 7.51–7.24 (m, 10H, Ph), 3.94 (d, *J* = 13.3 Hz, 2H, CH_2_Ph), 3.85–3.75 (m, 4H, H-4, H-5, 2 × H-7), 3.73–3.60 (m, 3H, H-6, CH_2_Ph), 3.47 (d, *J*_3,4_ = 3.1 Hz, 1H, H-3), 2.93 (d, *J*_1,1_ = 13.7 Hz, 1H, H-1), 2.77 (d, 1H, H-1); ^13^C NMR δ 139.8, 130.6, 130.3, 129.6 (Ph), 97.5 (C-2), 75.8, 74.2, 73.3, 68.7 (4C, C-3, C-4, C-5, C-6), 63.2 (C-7), 60.2 (2C, CH_2_Ph), 59.9 (C-1); anal. calcd for C_21_H_27_NO_6_: C, 64.77; H, 6.43; found: C, 63.07; H, 6.50.

**1-(*N-*(6-Hydroxyhexyl)amino)-1-deoxy-α-D-*manno*-hept-2-ulose (29):** Following general method A, the mixture of aldoheptoses **19** (360 mg) was treated with 6-aminohexanol (200 mg, 1.71 mmol, 1 equiv) in EtOH (5 mL) and 1,4-dioxane as co-solvent in the presence of acetic acid (0.1 mL, 1.71 mmol, 1 equiv) at 50 °C for three days. When TLC indicated complete consumption of the starting material, silica-gel chromatography (CHCl_3_/MeOH 4:1 v/v containing 10% of concd NH_4_OH) gave the respective Amadori compound **29** (360 mg) exclusively in the α-pyranose form in a yield of 70%. [α]_D_ = +7 (*c* 1.0, MeOH); ^1^H NMR (methanol-*d*_4_) δ 3.93–3.83 (m, 3H, H-3, H-5, H-7), 3.83–3.71 (m, 2H, H-6, H-7), 3.71–3.62 (m, 1H, H-4), 3.59 (t, 2H, H-13), 3.36 (d, 1H, H-1), 3.12 (d, *J*_1,1_ = 12.6 Hz, 1H, H-1), 3.04 (q, 2H, H-8), 1.81–1.69 (m, 2H, H-9), 1.64–1.53 (m, 2H, H-12), 1.51–1.40 (m, 4H, H-13, H-14); ^13^C NMR δ 96.3 (C-2), 75.2, 74.6, 72.6, 67.9 (4C, C-3, C-4, C-5, C-6), 62.8 (C-13), 62.6 (C-7), 55.0 (C-1), 49.4 (C-8), 33.6 (C-12), 27.5, 26.8, 26.5 (3C, C-9, C-10, C-11); anal. calcd for C_13_H_27_NO_7_: C, 50.50; H, 8.82; found: C, 50.45; H, 8.89.

**1-(*N*-(6-Hydroxyhexyl)amino)-1-*N*,2-*O*-carbonyl-1-deoxy-β-D-*manno*-pyranose (30):** General method C was applied to compound **29** (200 mg, 0.6 mmol) in H_2_O (5 mL) and 1,4-dioxane (1 mL), Na_2_CO_3_ (0.74 g, 6.9 mmol, 6 equiv) and triphosgene (0.35 g, 1.12 mmol, 1.8 equiv). Silica-gel chromatography (CHCl_3_/MeOH 6:1 v/v containing 1% of concd NH_4_OH) gave the corresponding cyclic carbamate **30** in the pure β-anomeric pyranoid form (160 mg) in a yield of 75%. β-Anomer: [α]_D_ = +22 (0.9, MeOH); ^1^H NMR (methanol-*d*_4_) δ 3.92 (d, *J*_3,4_ = 2.6 Hz, 1H, H-3), 3.83 (d, *J*_1,1_ = 10.7 Hz, 1H, H-1), 3.81–3.65 (m, 5H, H-4, H-5, H-6, 2 × H-7), 3.56 (t, 2H, H-13), 3.48 (d, 1H, H-1), 3.32–3.22 (m, 2H, H-8), 1.64–1.47 (m, 4H, H-9, H-12), 1.46–1.26 (m, 4H, H-10, H-11); ^13^C NMR δ 158.2 (C=O), 103.8 (C-2), 77.1, 73.2, 72.6, 67.3 (4C, C-3, C-4, C-5, C-6), 62.9 (C-13), 62.3 (C-7), 54.4 (C-1), 44.7 (C-8), 33.5 (C-12), 28.1, 27.3, 26.5 (3C, C-9, C-10, C-11); anal. calcd for C_14_H_25_NO_8_: C, 50.17; H, 7.53; found: C, 50.13; H, 7.60.

**1-(*N*-(5-(Methoxycarbonyl)pentyl)amino)-1-deoxy-α-D-*manno*-hept-2-ulopyranose (31):** Following general method B, aldose **19** (350 mg, 1.7 mmol) was treated with a mixture of 6-aminohexanoic acid methyl ester hydrochloride (600 mg, 3.4 mmol, 2.0 equiv) in EtOH (5 mL) and H_2_O (1 mL) as co-solvent containing Et_3_N (480.0 μL, 3.4 mmol, 2.0 equiv), and the reaction mixture was stirred at 40 °C for four days. The solvents were removed under reduced pressure and silica-gel chromatography (CHCl_3_/MeOH 3:1 v/v containing 1 % of concd NH_4_OH) gave 600 mg of pyranose **31** in a yield of 90% as a yellow oil. [α]_D_ = +8 (*c* 1.2, MeOH); ^1^H NMR (methanol-*d*_4_) δ 3.99–3.72 (m, 6H, H-3, H-4, H-5, H-6, 2 × H-7), 3.68 (s, 3H, OCH_3_), 3.38 (d, 1H, *J*_1,1_ = 12.3 Hz, H-1), 3.18 (d, 1H, H-1), 3.06 (bt, 2H, H-8), 2.39 (t, 2H, H-12), 1.84–1.62 (m, 4H, H-9, H-11), 1.51–1.39 (m, 2H, H-10); ^13^C NMR δ 175.8 (C-13), 96.2 (C-2), 75.2, 74.6, 72.5, 67.8 (4C, C-3, C-4, C-5, C-6), 62.5 (C-7), 55.1 (C-1), 52.1 (OCH_3_), 47.7 (C-8), 34.4 (C-12), 27.1, 26.4, 25.5 (3, C-9, C-10, C-11); anal. calcd for C_14_H_27_NO_8_: C, 49.87; H, 8.09; found: C, 49.78; H, 8.12.

**1-(*N*-(5-(Methoxycarbonyl)pentyl)amino)-1-*N*,2-*O*-carbonyl-1-deoxy-β-D-*manno*-hept-2-ulopyranose (32):** General method C was applied to Amadori product **31 (**200 mg, 0.6 mmol), Na_2_CO_3_ (680.0 mg, 6.4 mmol, 6.0 equiv) and triphosgene (320 mg, 1.1 mmol, 1.8 equiv) in H_2_O (7 mL) and 1,4-dioxane as co-solvent (1 mL). After complete consumption of the starting material, the solvent was removed under reduced pressure, and silica-gel chromatography (CHCl_3_/MeOH 6:1 v/v containing 1% of concd NH_4_OH) gave cyclic carbamate **32** (110 mg, 50%) in the β-anomeric pyranoid form exclusively as a slightly yellow oil. [α]_D_ = +46 (*c* 1.4, MeOH); ^1^H NMR (methanol-*d*_4_) δ 3.89–3.83 (m, *J*_3,4_ = 2.7 Hz, 2H, H-3, H-7), 3.81 (d, *J*_1,1_ = 10.4 Hz, 1H, H-1), 3.79–3.69 (m, 4H, H-4, H-5, H-6, H-7), 3.68 (s, 3H, OCH_3_), 3.47 (d, 1H, H-1), 3.36–3.30 (m, 2H, H-8), 2.38 (t, 2H, H-12), 1.74–1.55 (m, 4H, H-9, H-11), 1.44–1.30 (m, 2H, H-10); ^13^C NMR δ 175.9 (C-13), 158.1 (NCO), 103.8 (C-2), 77.2, 72.7, 72.6, 67.5 (4C, C-3, C-4, C-5, C-6), 62.6 (C-7), 54.3 (C-1), 52.1 (OCH_3_), 44.5 (C-8), 34.6 (C-12), 27.9, 27.0, 25.6 (3C, C-9, C-10, C-11); anal. calcd for C_15_H_25_NO_9_: C, 49.61; H, 6.95. found: C, 49.56; H, 6.98.

**1-(*N*,*N*-Dibenzylamino)-3-acetamido-1,2-dideoxy-α-D-*gluco*-hept-2-ulopyranose (33a) and -furanose (33b):** General method for sugar elongation was applied to GlcNAc **20** (3 g, 13.6 mmol) dissolved in pyridine (45 mL, 0.56 mmol) and triethylamine (0.15 mL, 1.1 mmol, 12.6 equiv) employing HCN (4.5 mL, 0.11 mmol, 8.5 equiv). After four days complete consumption of the starting material was indicated by TLC (per-*O*-acetylation of a small sample, C/EE 1:1 v/v) and 3.5 g of the crude heptononitriles **21a** and **21b** were obtained. Heptononitriles **21a** and **21b** (3.5 g, 14.1 mmol) were treated as described in distd water (40 mL) employing acetic acid (1.23 mL, 1.5 equiv) and Pd/BaSO_4_ (2.1 g). After TLC (CHCl_3_/MeOH 1:1 v/v containing 0.25% concd NH_4_OH) showed complete consumption of the starting material, the workup was applied as described, and 2.8 g of a mixture of 3-acetamido-3-deoxy-D-*gluco*-D-*ido*/D-*gulo*-heptoses **22a** and **22b** were obtained, which were used for the Amadori rearrangement immediately without any further purification. General procedure A was applied to aldoheptoses **22** (300 mg, 1.2 mmol) in EtOH (6 mL) employing dibenzylamine (460 mL, 2.4 mmol, 2 equiv) and acetic acid (120 mL, 2.1 mmol, 1.8 equiv). The reaction mixture was stirred at 40 °C for three days. When TLC indicated complete consumption of the starting material the solvent was removed under reduced pressure. Column chromatography (CHCl_3_/MeOH 3:1 v/v containing 1% concd NH_4_OH) gave 210 mg of the pyranoid product **33a** and a minor amount of furanoid compound **33b** in an overall yield of 41%. α-Anomer: ^1^H NMR (methanol-*d*_4_) δ 7.39–7.20 (m, 10H, Ph), 4.02 (d, 1H, *J*_3,4_ = 9.6 Hz, H-3), 4.06 (d, 2H, *J* = 13.4 Hz, CH_2_Ph), 3.71–3.44 (m, 4H, H-6, 2 × H-7, H-5), 3.27 (dd, *J*_4,5_ = 8.3 Hz, 1H, H-4), 3.26 (d, 2H, CH_2_PH), 2.51–2.41 (2 × d, *J*_1,1_ = 14.2 Hz, 2H, 2 × H-1), 1.46 (s, 3H, CH_3_); ^13^C NMR δ 173.7 (NHCO), 140.1, 130.5, 129.6, 128.5 (Ph), 98.3 (C-2), 74.4, 73.8, 72.2 (3C, C-3, C-4, C-5, C-6), 63.0 (C-7), 60.5 (2C, CH_2_Ph), 57.4 (C-1), 56.5 (C-3); anal. calcd for C_23_H_30_N_2_O_6_: C, 64.17; H, 7.04; found: C, 64.12; H, 7.09.

**1-(*N*-(6-Hydroxyhexyl)amino)-3-acetamido-1,2-dideoxy-α-D-*gluco*-hept-2-ulopyranose (34):** Following general method A, the mixture of 3-NHAc-aldoheptoses **22** (**a** and **b**) (300 mg, 1.2 mmol) was treated with 6-aminohexanol (220 mg, 1.9 mmol, 1.6 equiv) in EtOH (6 mL) in the presence of acetic acid (0.26 mL, 4.54 mmol, 3.8 equiv) at 60 °C for two days. When TLC indicated complete consumption of the starting material, the solvent was removed under reduced pressure. Silica-gel chromatography (CHCl_3_/MeOH 5:1 v/v containing 30% of concd NH_4_OH) gave the respective Amadori compound as α-pyranose **34** (210 mg) in a yield of 50%. α-Anomer: [α]_D_ = +19 (*c* 3.1, MeOH); ^1^H NMR (methanol-*d*_4_) δ 3.83–3.75 (m, 5H, H-3, H-4, H-5, 2 × H-7), 3.58 (t, 2H, H-13), 3.47 (ddd, *J*_5,6_ = 7.8 Hz, *J*_6,7’_ = 2.4 Hz, *J*_6,7_ = 8.9 Hz, 1H, H-6), 2.79 (d, *J*_1,1_ = 12.3 Hz, 1H, H-1), 2.67 (t, 2H, H-8), 2.61 (d, 1H, H-1), 2.05 (s, 3H, CH_3_), 1.45–1.36 (m, 4H, H-10, H-11); ^13^C NMR δ 174.6 (NHCO), 98.1 (C-2), 74.2, 73.4, 71.7 (3C, C-4, C-5, C-6), 62.9 (C-13), 62.5 (C-7), 56.0 (2C, C-1, C-3), 50.9 (C-8), 33.6 (C-12), 29.9, 28.0, 26.8 (3C, C-9, C-10, C-11), 22.7 (CH_3_); anal. calcd for C_15_H_30_N_2_O_7_: C, 51.41; H, 8.64; found: C, 51.36; H, 8.68.

**1-(*N*-(5-(Methoxycarbonyl)pentyl)amino)-2-acetamido-1,2-dideoxy-α-D-*gluco*-hept-2-ulopyranose (35):** Following general method B, a mixture of 6-aminohexanoic acid methyl ester hydrochloride (350 mg, 1.9 mmol, 1.6 equiv) in EtOH (6 mL) containing Et_3_N (260.0 μL, 1.9 mmol, 1.6 equiv) was applied to the mixture of 3-NHAc-aldoses **22** (**a** and **b**) (300 mg, 1.2 mmol), and the reaction mixture was stirred at 40 °C for four days. The solvents were removed under reduced pressure, and silica-gel chromatography (CHCl_3_/MeOH 5:1 v/v containing 1% of concd NH_4_OH) gave 115 mg of 3-NHAc-pyranose **35** in a yield of 25% (115 mg) as a yellow oil. [α]_D_ = +6 (*c* 0.9, MeOH); ^1^H NMR (methanol-*d*_4_) δ 3.92–3.77 (m, 4H, H-3, H-4, 2 × H-7), 3.74 (d, *J*_2,3_ = 10.3 Hz, 1H, H-3), 3.69 (s, 3H, OCH_3_), 3.50 (dd, *J*_5,6 = 5,4_ = 9.0 Hz, 1H, H-5), 3.15–2.93 (m, 4H, 2 × H-1, 2 × H-8), 2.58 (t, 2H, H-12), 2.1 (s, 3H, COCH_3_), 1.86–1.63 (m, 4H, H-9, H-11), 1.54–1.41 (m, 2H, H-10); ^13^C NMR δ 175.7, 175.5 (2C, NHCO, C-13), 96.7 (C-2), 74.5, 72.1, 71.4 (3C, C-4, C-5, C-6), 61.7 (C-7), 56.0 (C-3), 54.1 (C-1), 52.1 (OCH_3_), 49.5 (C-8), 34.4 (C-13), 26.9, 26.7, 25.4 (3C, C-9, C-10, C-11), 22.5 (CH_3_); anal. calcd for C_16_H_30_N_2_O_8_: C, 50.78; H, 8.00; found: C, 50.83; H, 8.06.

**1-*N*-[(5*S*)-(*tert*-Butoxycarbonylamino)-5-(methoxycarbonyl)pentyl]amino-3-acetamido-1,2-dideoxy-α-D-*gluco*-hept-2-ulopyranose (36):** Following general method B, a mixture of Boc-Lys-OMe [36] (360 mg, 1.2 mmol, 1.0 equiv) in EtOH (6 mL) and acetic acid (70.0 μL, 1.2 mmol, 1.0 equiv) was applied to the mixture of 3-NHAc-aldoses **22** (**a** and **b**) (300 mg, 1.2 mmol), and the reaction mixture was stirred at 50 °C for two days. After complete conversion of the starting material, the solvents were removed under reduced pressure, and silica-gel chromatography (CHCl_3_/MeOH 7:1 v/v containing 10% of concd NH_4_OH) gave 3-NHAc-pyranose **36** in a yield of 30% (180 mg) as a yellow oil. [α]_D_ = +12 (*c* 0.4, MeOH); ^1^H NMR (methanol-*d*_4_) δ 4.17–4.07 (m, 2H, H-12), 3.58–3.69 (m, 8H, H-3, H-4, H-6, 2 × H-7, OCH_3_), 3.48 (dd, *J*_4,5 = 5,6_ = 8.6 Hz, 1H, H-5), 2.75 (d, *J*_1,1_ = 12.2 Hz, 1H, H-1), 2.61 (t, 2H, H-8), 2.53 (d, 1H, H-1), 2.05 (s, 3H, COCH_3_), 1.87–1.63 (m, 4H, H-10, H-11), 1.63–1.49 (m, 2H, H-9), 1.46 (s, 9H, 3 × CH_3_); ^13^C NMR δ 175.1, 174.1, 158.2 (3C, 3 × C=O), 98.3 (C-2), 80.6 (C-13), 74.1, 73.7, 71.8 (3C, C-3, C-4, C-5), 62.6 (C-7), 56.3 (C-1), 55.9 (C-9), 55.1 (C-12), 52.7 (OCH_3_), 50.7 (C-8), 32.5, 29.9 (2C, C-10, C-11), 28.8 (3C, 2 × CH3), 24.5 (C-9), 22.7 (OCH_3_); anal. calcd for C_21_H_39_N_3_O_10_: C, 51.11; H, 7.99; found: C, 51.16; H, 8.06.

## Supporting Information

File 1 NMR data of compounds **16**, **19**, **23**–**36**.
