# Supplementary material for: The Amadori rearrangement as glycoconjugation method: Synthesis of non-natural C-glycosyl type glycoconjugates
Source: Beilstein J Org Chem. 2012 Sep 25;8:1619–29. doi: 10.3762/bjoc.8.185 (PMC3510994; doi:10.3762/bjoc.8.185)

Supporting Information

for

The Amadori rearrangement as glycoconjugation method: Synthesis of non-natural *C*-glycosyl type glycoconjugates

Katharina Gallas¶, Gerit Pototschnig¶, Florian Adanitsch, Arnold E. Stütz and Tanja M. Wrodnigg*

Address: Glycogroup, Institute of Organic Chemistry, Technical University Graz, Stremayrgasse 9, A-8010 Graz, Austria

Email: Tanja M. Wrodnigg – t.wrodnigg@tugraz.at

¶ These authors contributed equally to this work.

* Corresponding author

NMR data of compounds 16, 19, 23–36.

General

NMR spectra were recorded on a Bruker Ultrashield spectrometer at 300.36 and 75.53 MHz, respectively in methanol-d_4 or D_2O as indicated. Chemical shifts are listed in delta employing residual, non-deuterated solvent as the internal standard. Structures of crucial intermediates were unambiguously assigned by 1D-TOCSY and HSQC experiments.

D-*glycero*-L-*manno*/L-*gluco*-heptopyranose 16a and 16b ^{1}H, 300 MHz, D_2O

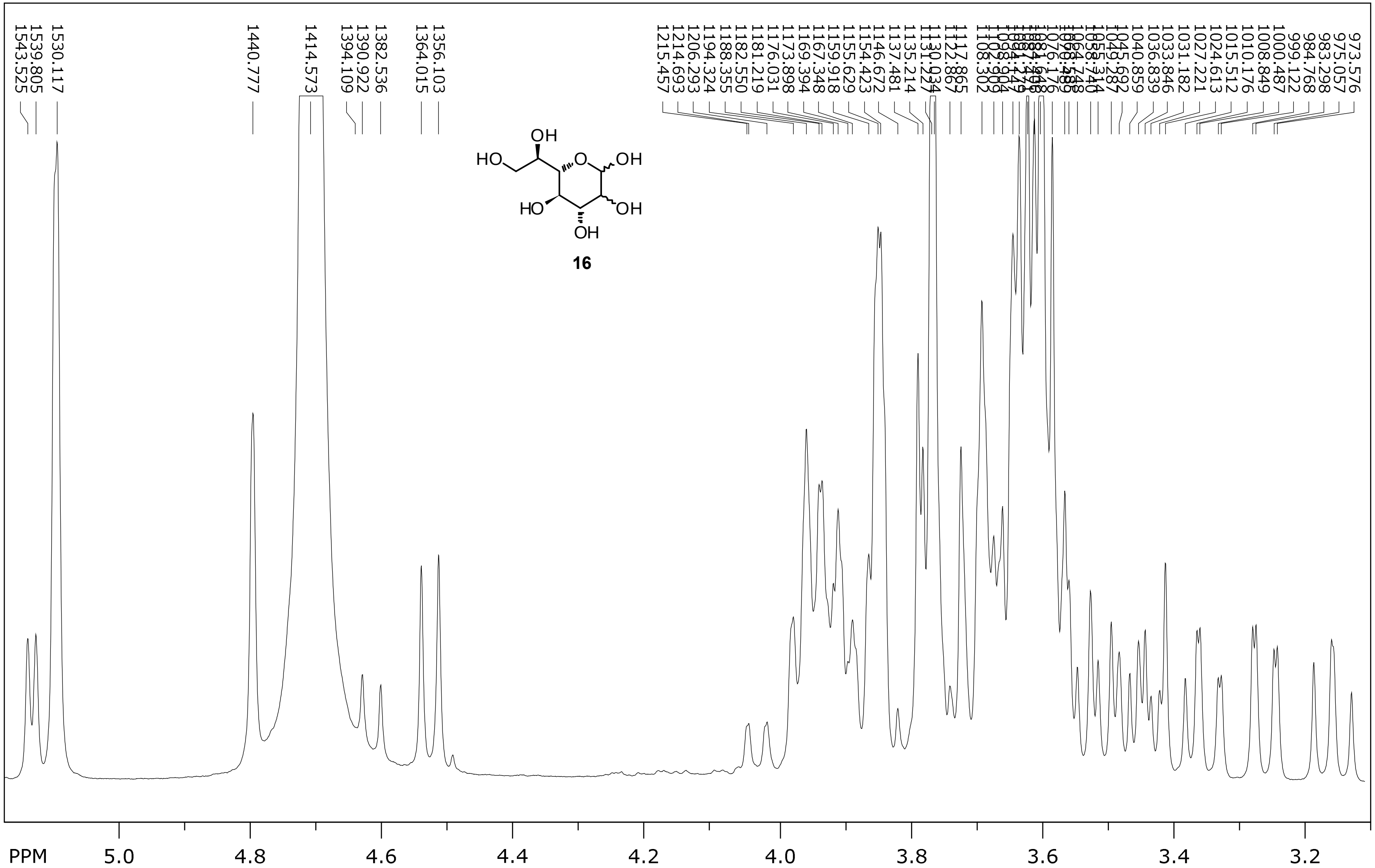

D-*glycero*-L-*manno*/L-*gluco*-heptopyranose 16a and 16b ^{13}C, 75 MHz, D_2O

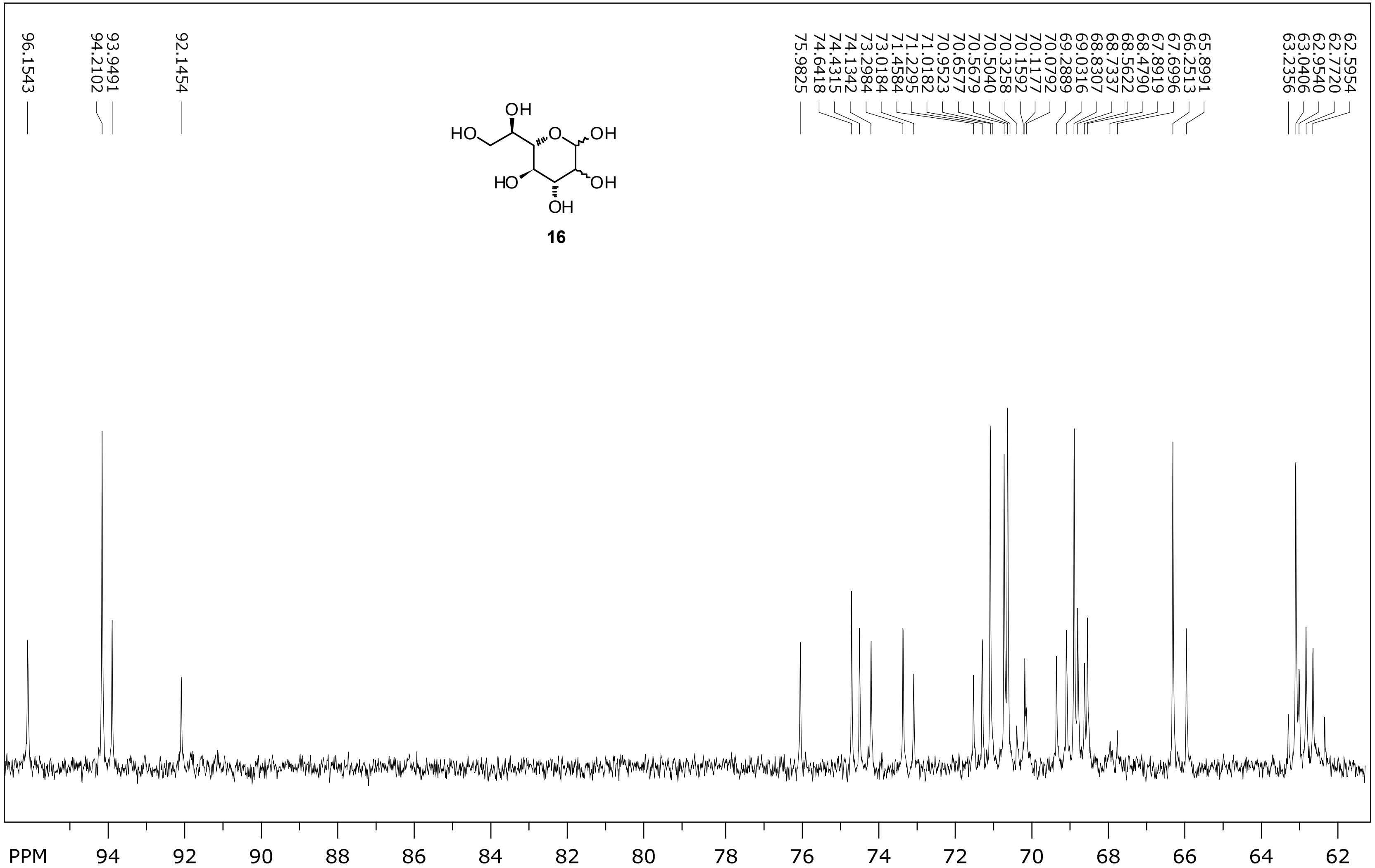

1-(*N*,*N*-Dibenzylamino)-1-deoxy-α-D-*galacto*-hept-2-ulopyranose (23a) and -furanose (23b), ^{1}H, 300 MHz, MeOH-d_4

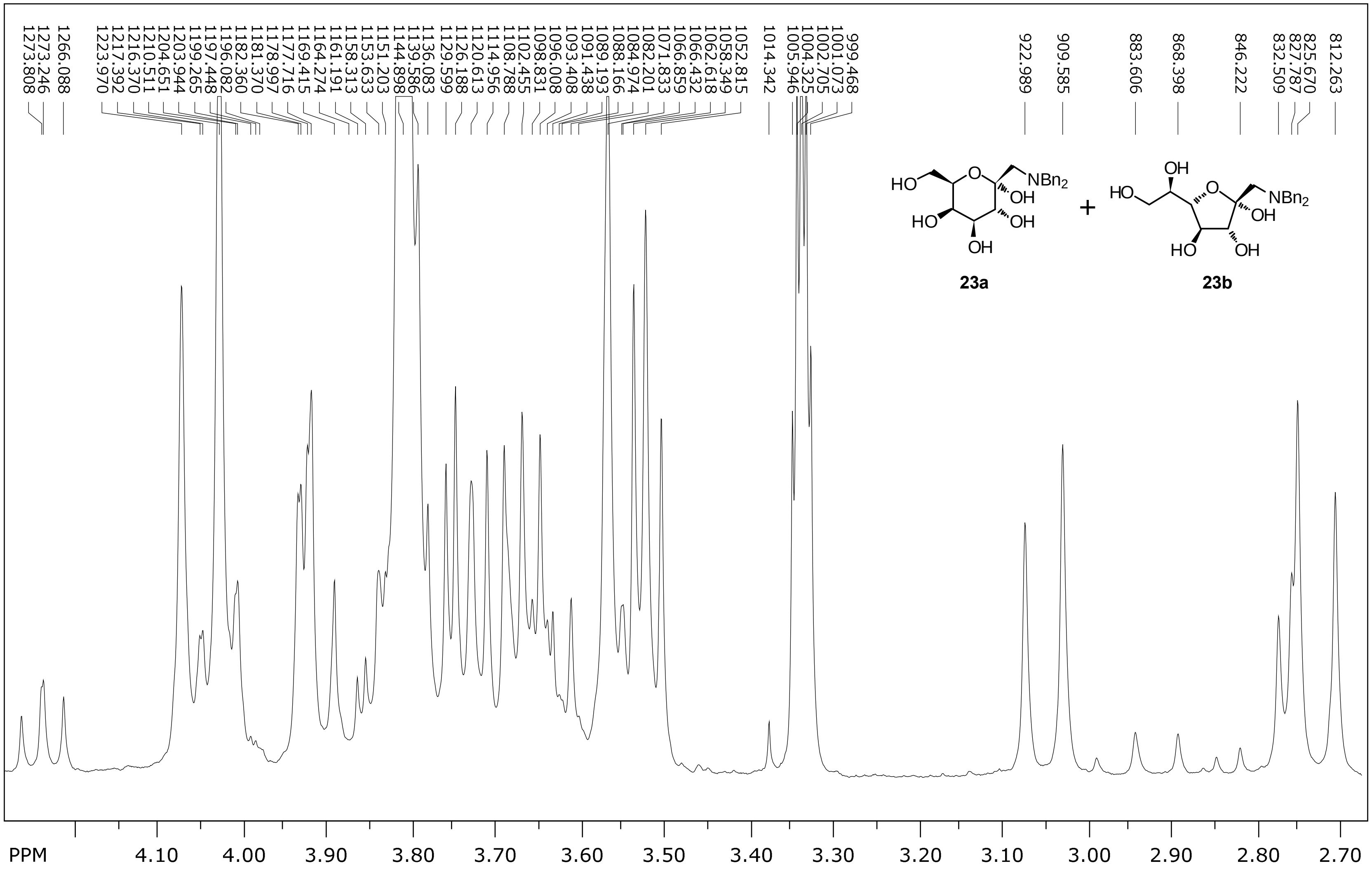

1-(*N*,*N*-Dibenzylamino)-1-deoxy-α-D-*galacto*-hept-2-ulopyranose (23a) and -furanose (23b), ^{13}C, 75 MHz, MeOH-d_4

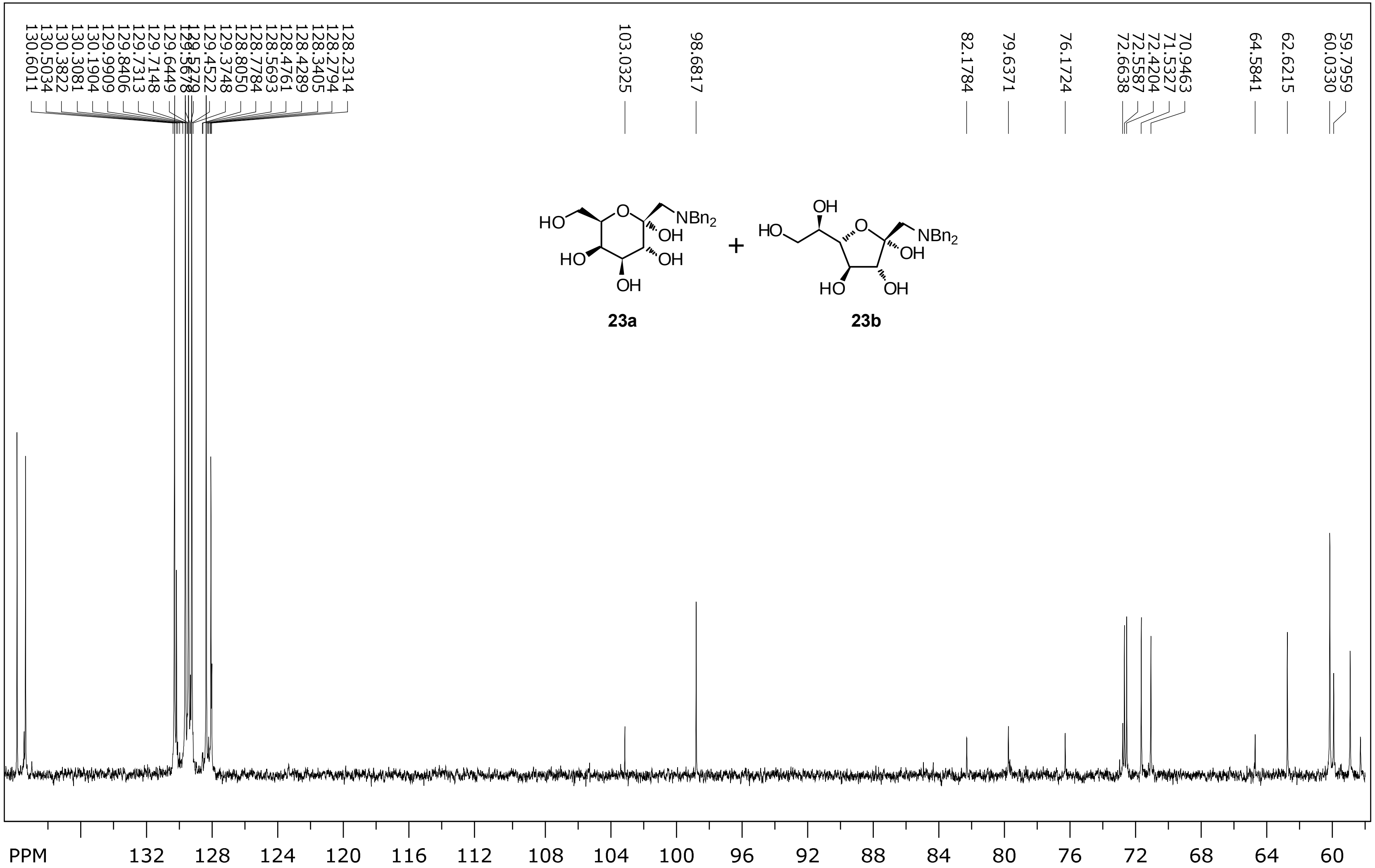

1-(*N*-(6-Hydroxyhexyl)amino)-1-deoxy-α-d-*galacto*-hept-2-ulopyranose (24a) and –furanose (24b) ^{1}H, 300 MHz, MeOH-d_4

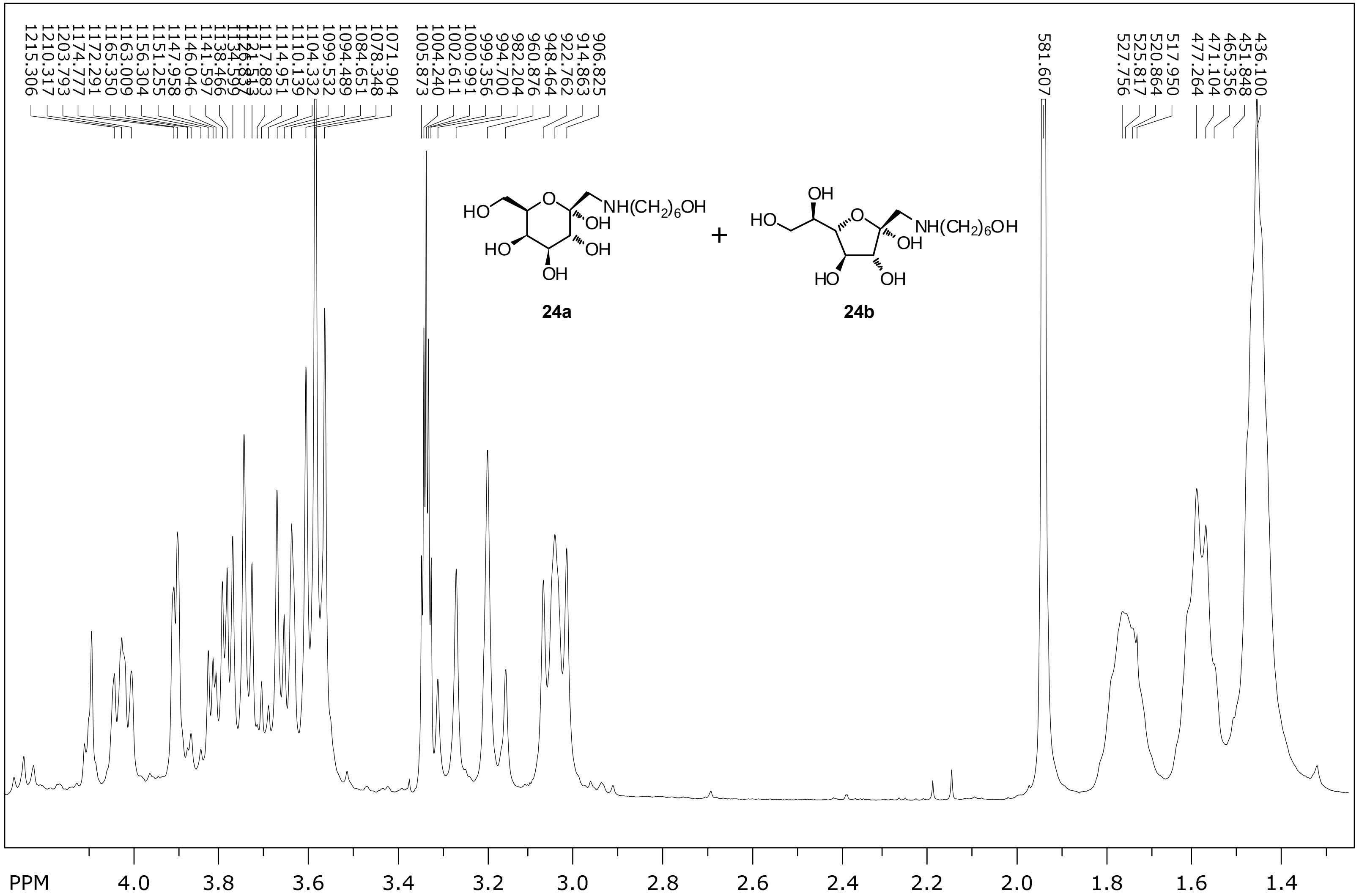

1-(*N*-(6-Hydroxyhexyl)amino)-1-deoxy-α-d-*galacto*-hept-2-ulopyranose (24a) and –furanose (24b) ^{13}C, 75 MHz, MeOH-d_4

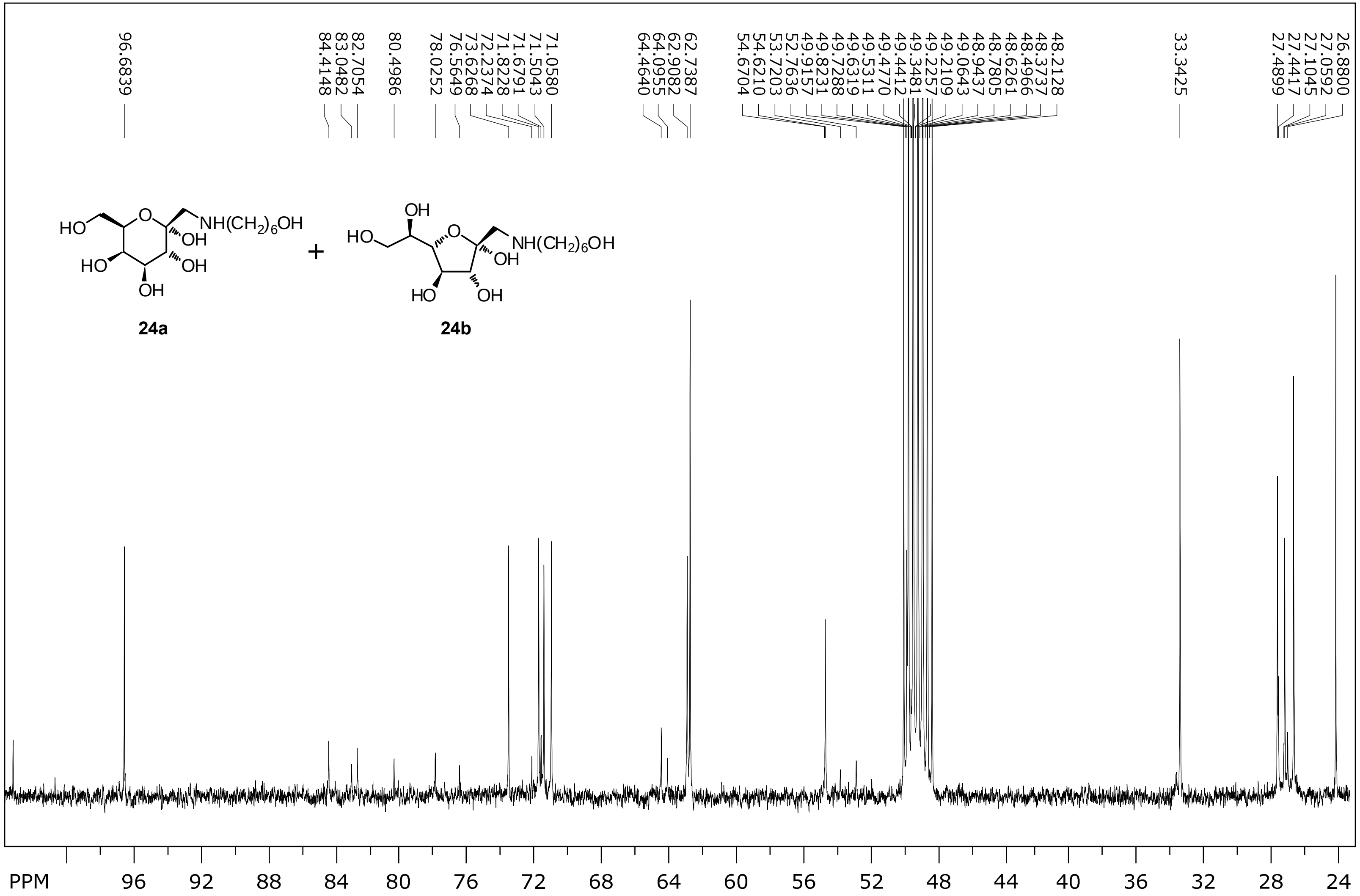

1-(*N*-(6-Hydroxyhexyl)amino)-1-*N*,2-*O*-carbonyl-1-deoxy-β-D-*galacto*-hept-2-ulopyranose 26a and -furanose 26b ^{1}H, 300 MHz, MeOH-d_4

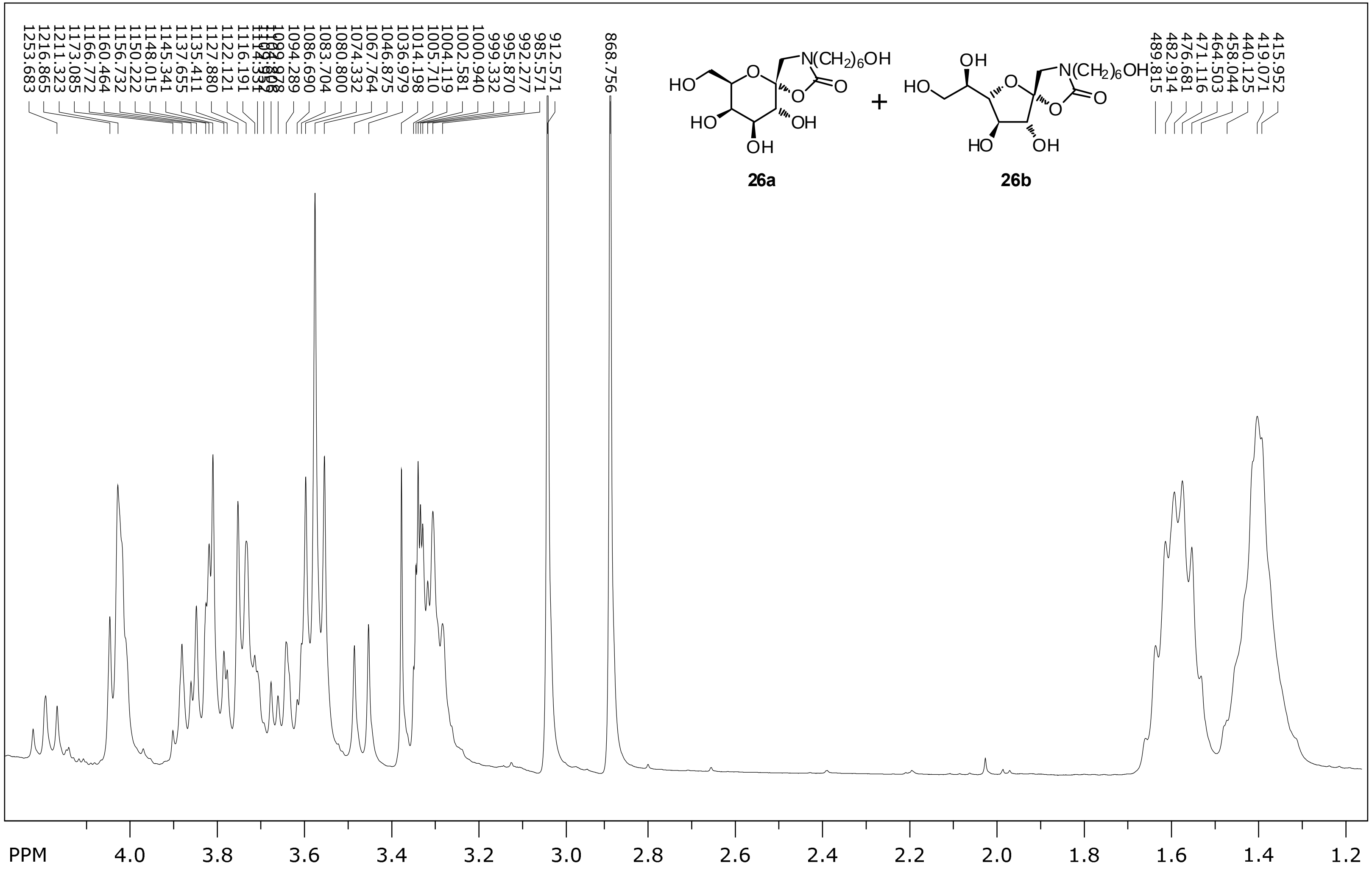

1-(*N*-(6-Hydroxyhexyl)amino)-1-*N*,2-*O*-carbonyl-1-deoxy-β-D-*galacto*-hept-2-ulopyranose 26a and -furanose 26b ^{13}C, 75 MHz, MeOH-d_4

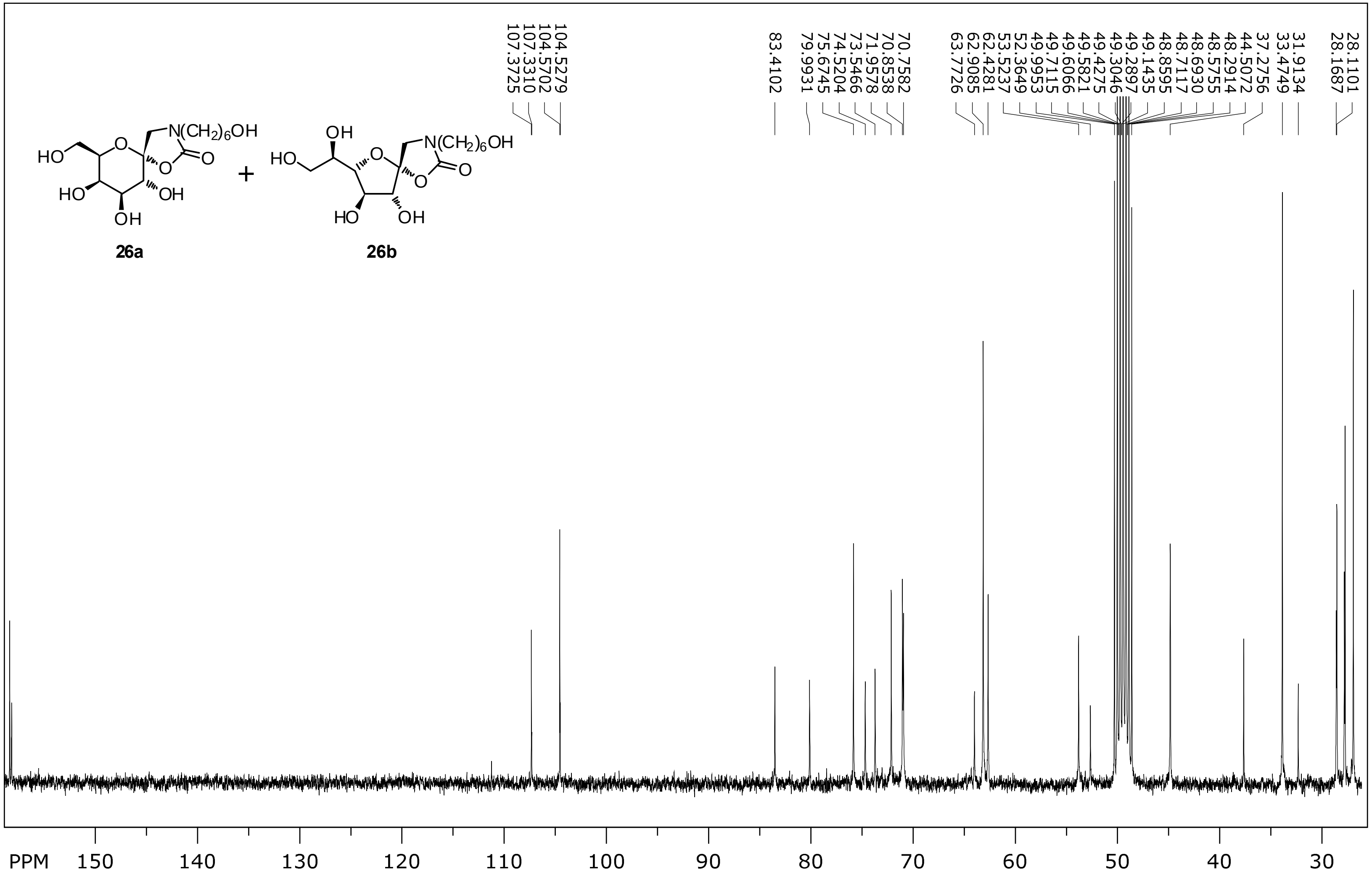

1-(*N*-(5-(Methoxycarbonyl)pentyl)amino)-1-deoxy-α-D-*galacto*-hept-2-ulopyranose (25a) and –furanose (25b) ^{1}H, 300 MHz, MeOH-d_4

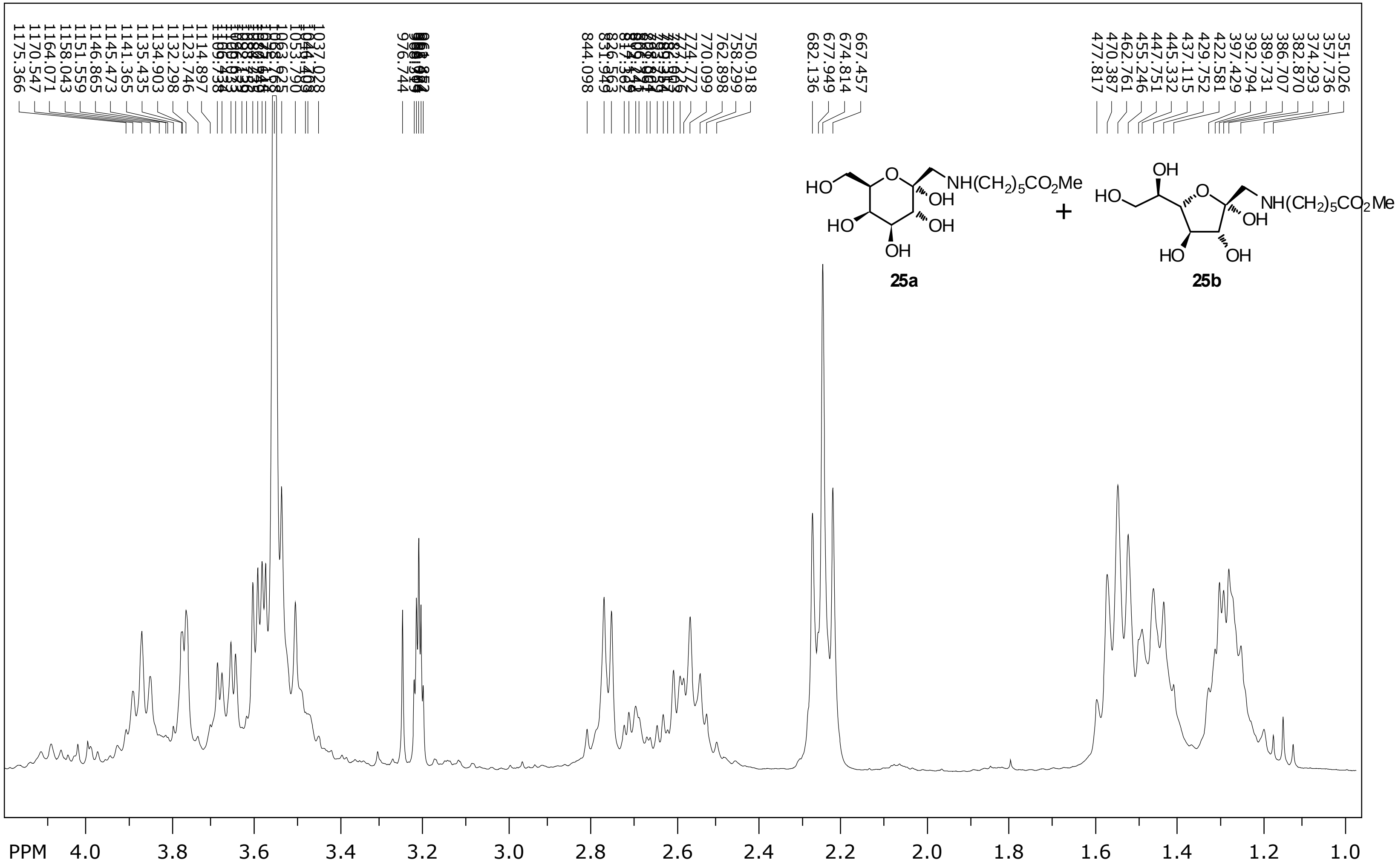

1-(*N*-(5-(Methoxycarbonyl)pentyl)amino)-1-deoxy-α-D-*galacto*-hept-2-ulopyranose (25a) and –furanose (25b) ^{13}C, 75 MHz, MeOH-d_4

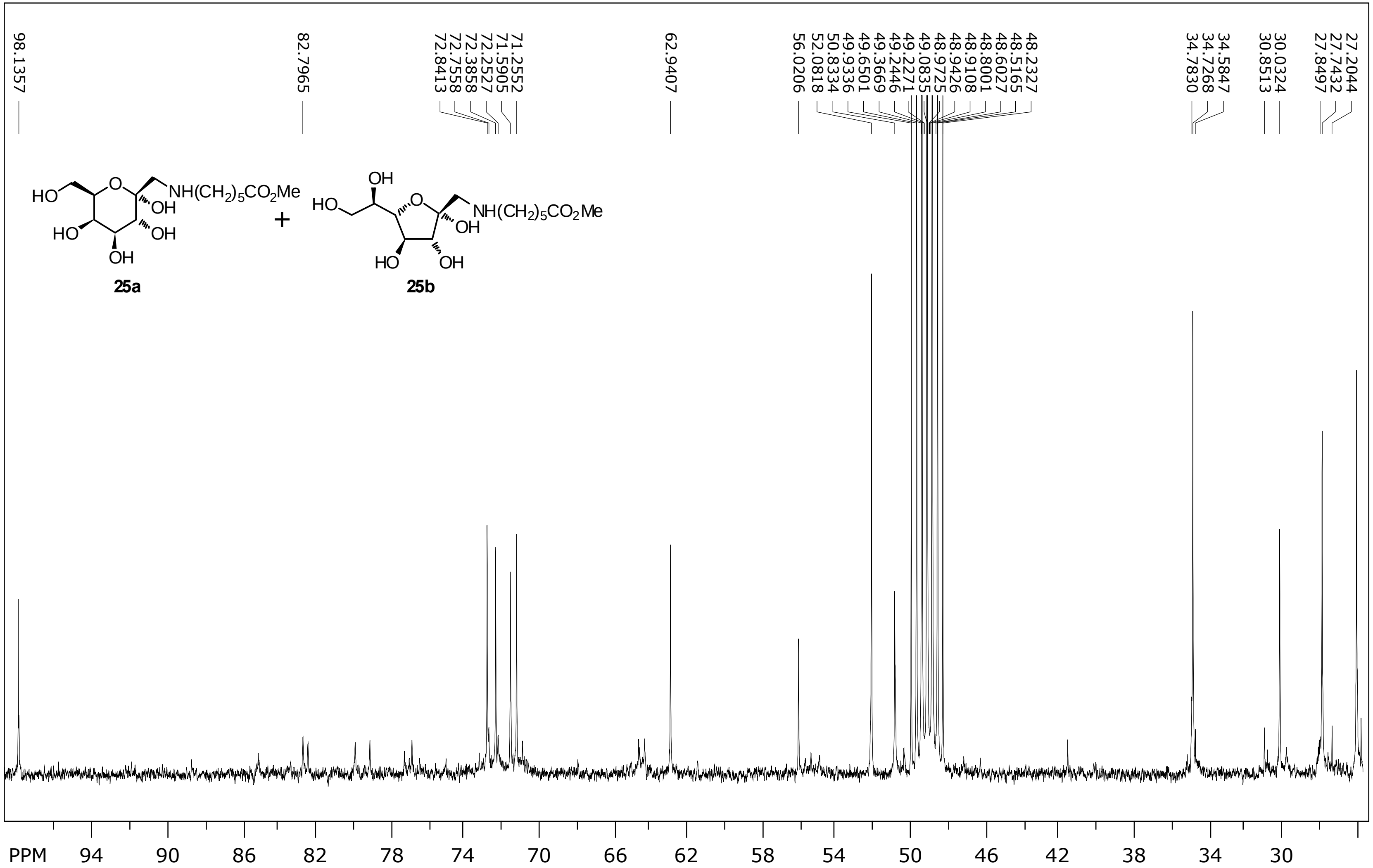

1-(*N*-(5-(Methoxycarbonyl)pentyl)amino)-1-*N*,2-*O*-carbonyl-1-deoxy-β-D-*galacto*-hept-2-ulopyranose (27a) and –furanose (27b) ^{1}H, 300 MHz, MeOH-d_4

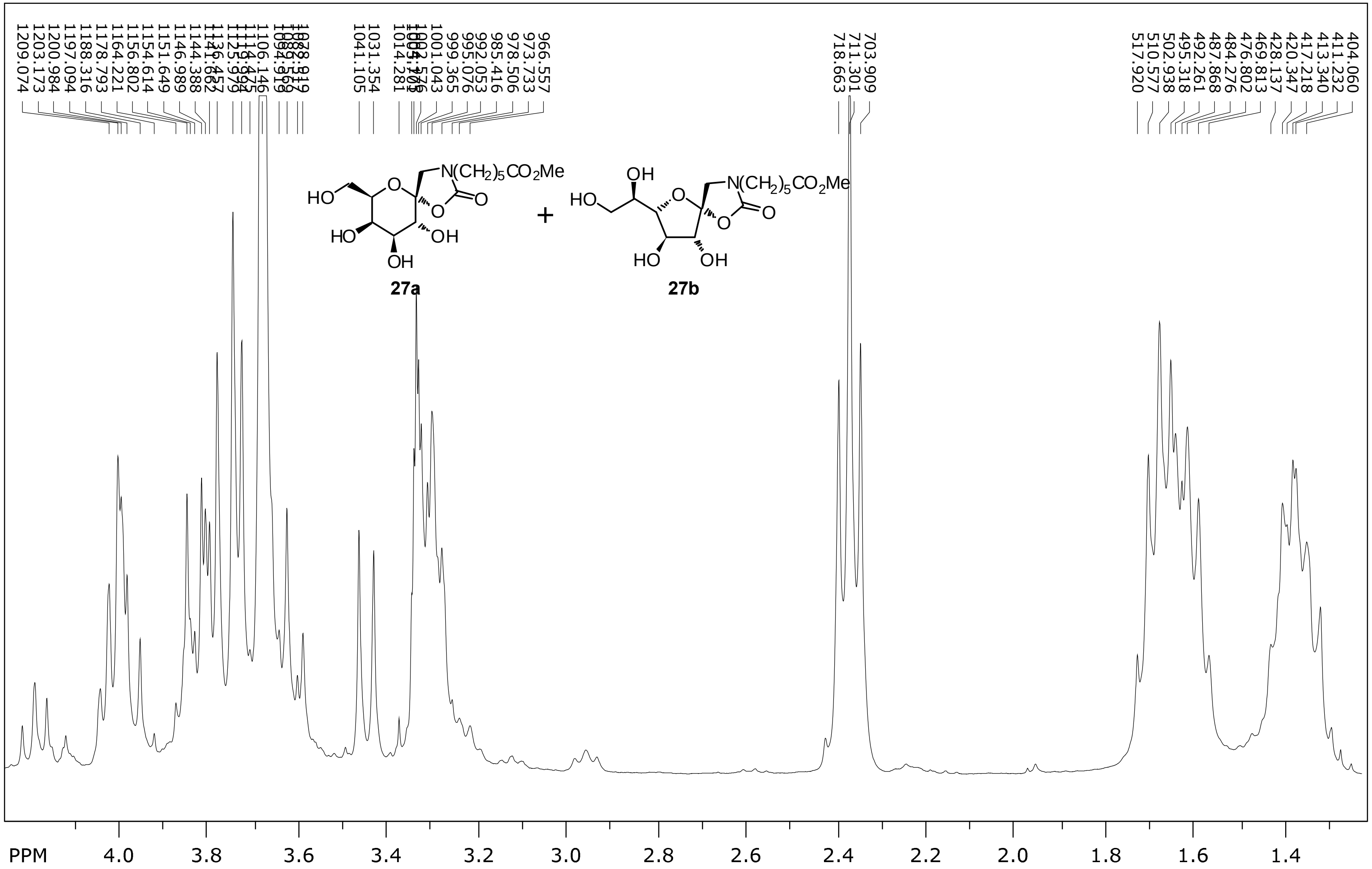

1-(*N*-(5-(Methoxycarbonyl)pentyl)amino)-1-*N*,2-*O*-carbonyl-1-deoxy-β-D-*galacto*-hept-2-ulopyranose (27a) and –furanose (27b) ^{13}C, 75 MHz, MeOH-d_4

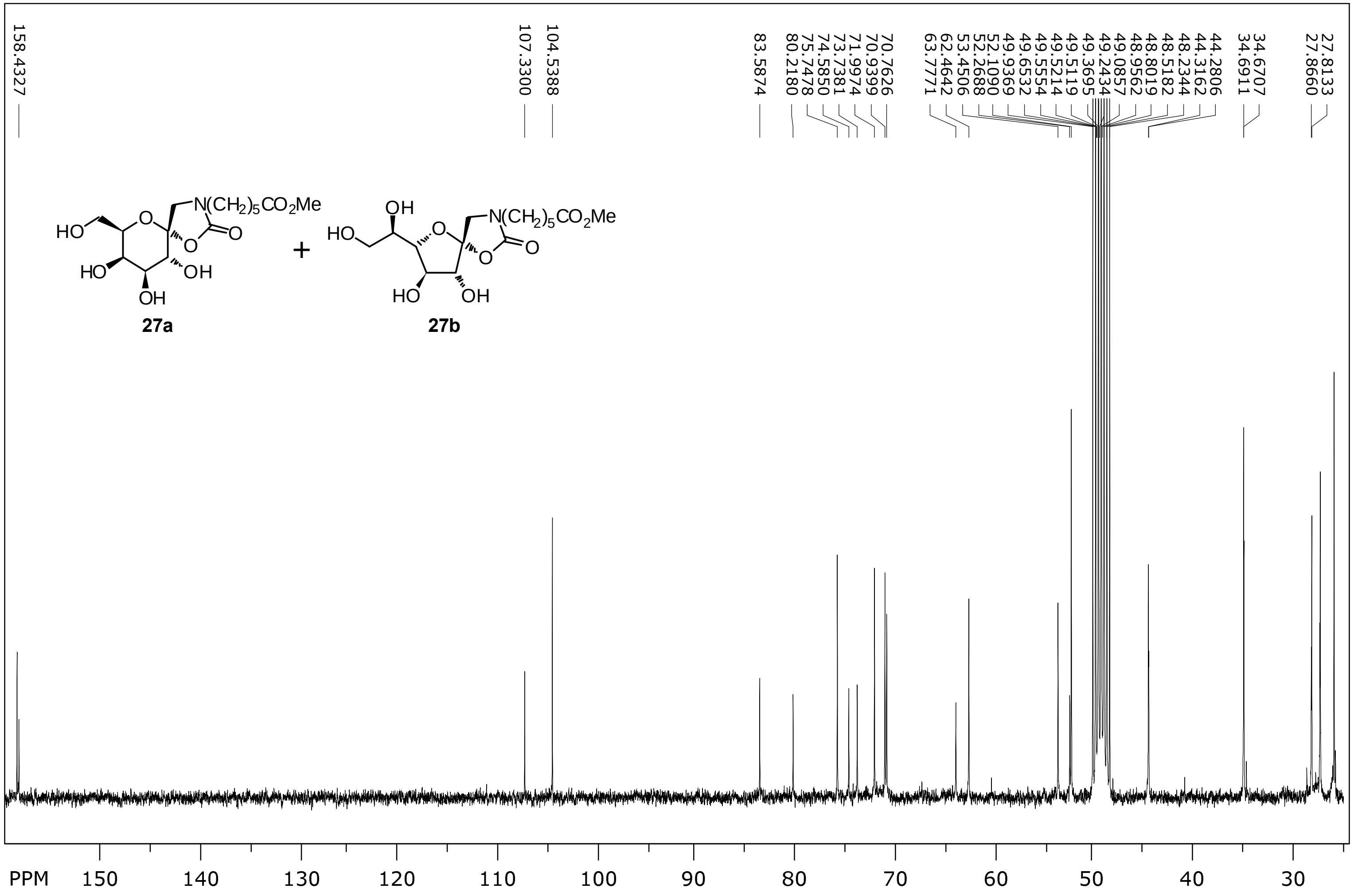

D-*glycero*-D-*galacto*/D-*talo*-heptopyranose 19a and 19b ^{1}H, 300 MHz, D_2O

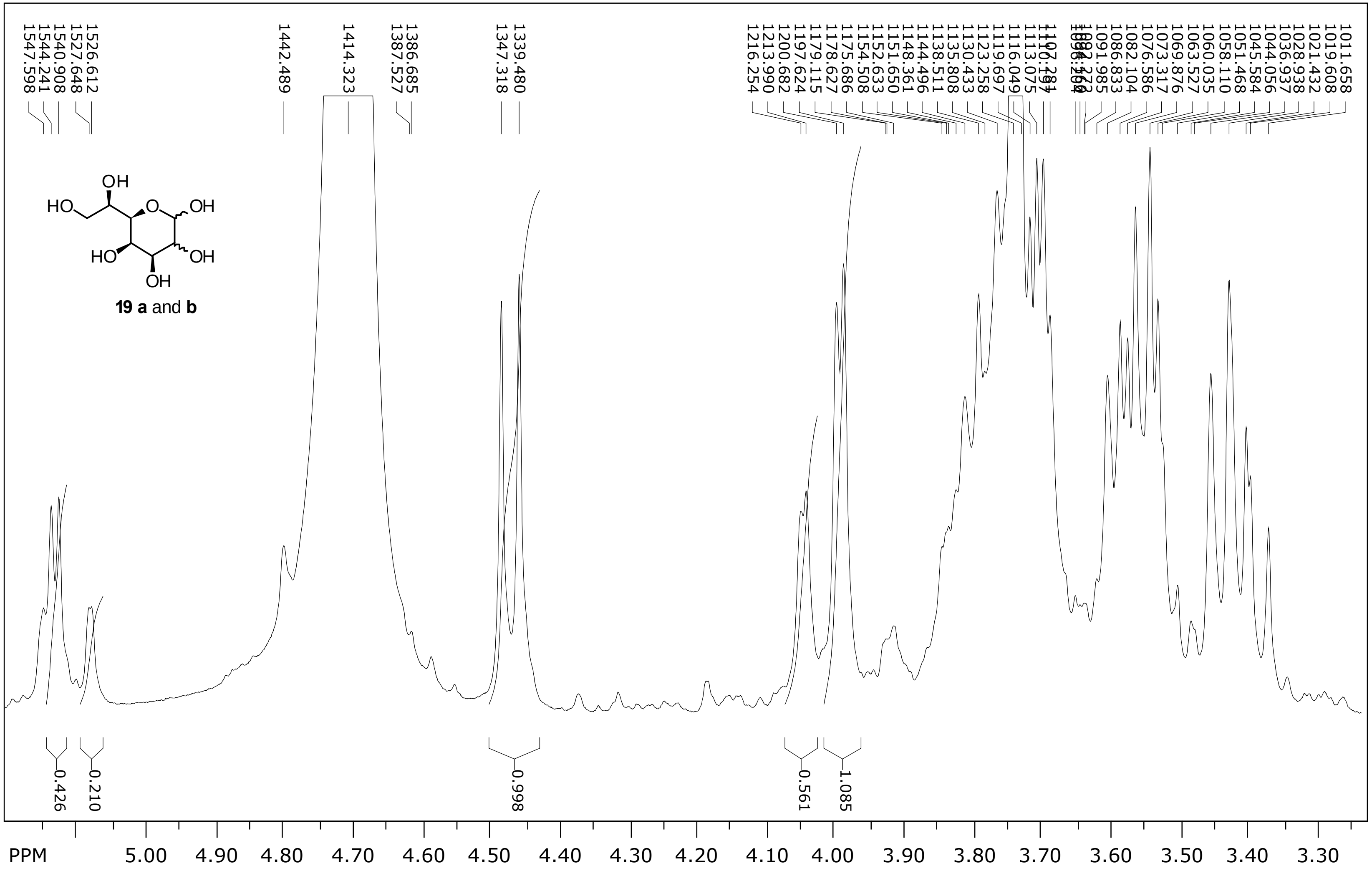

D-*glycero*-D-*galacto*/D-*talo*-heptopyranose 19a and 19b ^{13}C, 75 MHz, D_2O

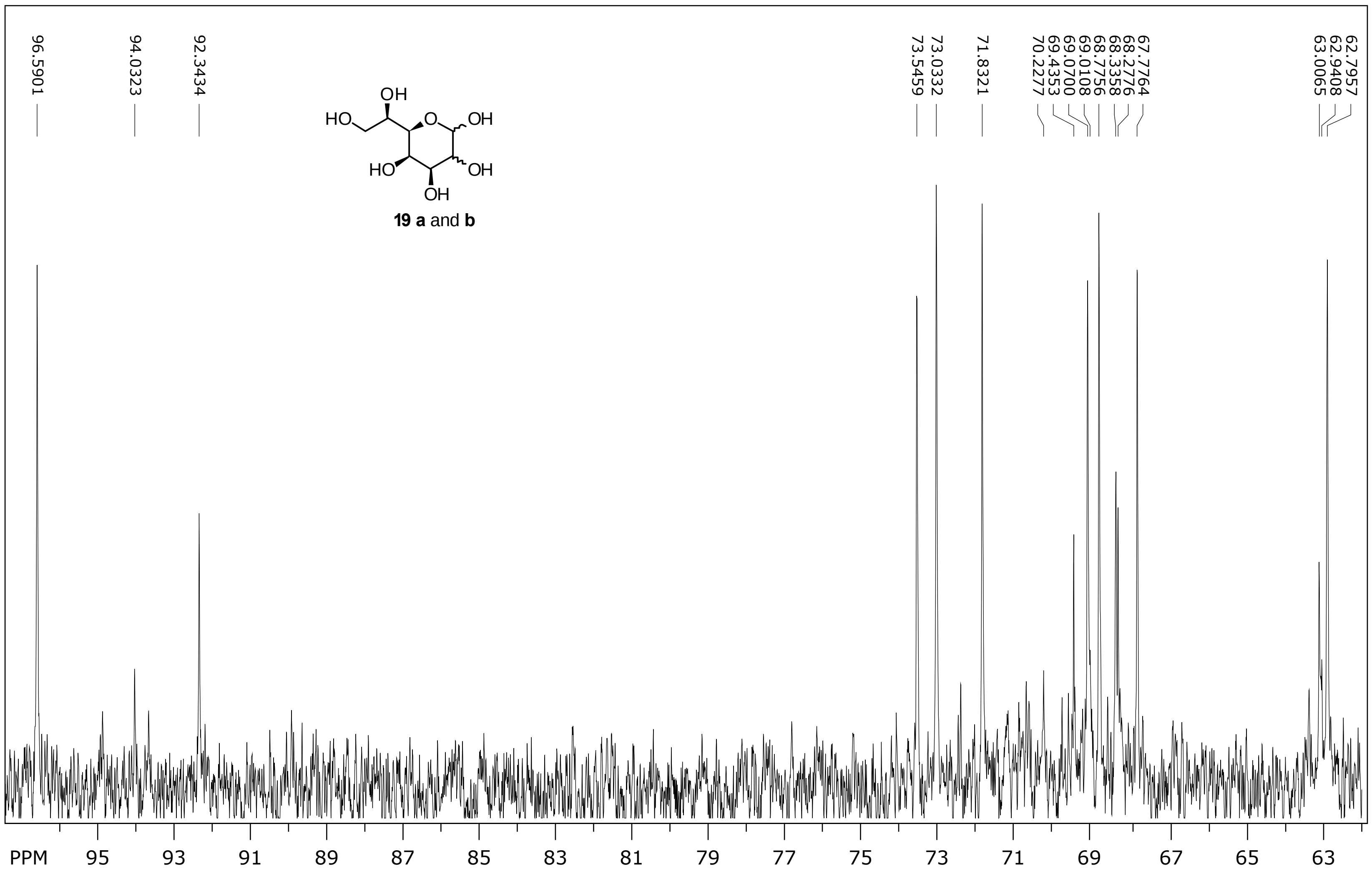

1-(*N*,*N*-Dibenzylamino)-1-deoxy-α-D-*manno*-hept-2-ulopyranose (28) ^{1}H, 300 MHz, MeOH-d_4

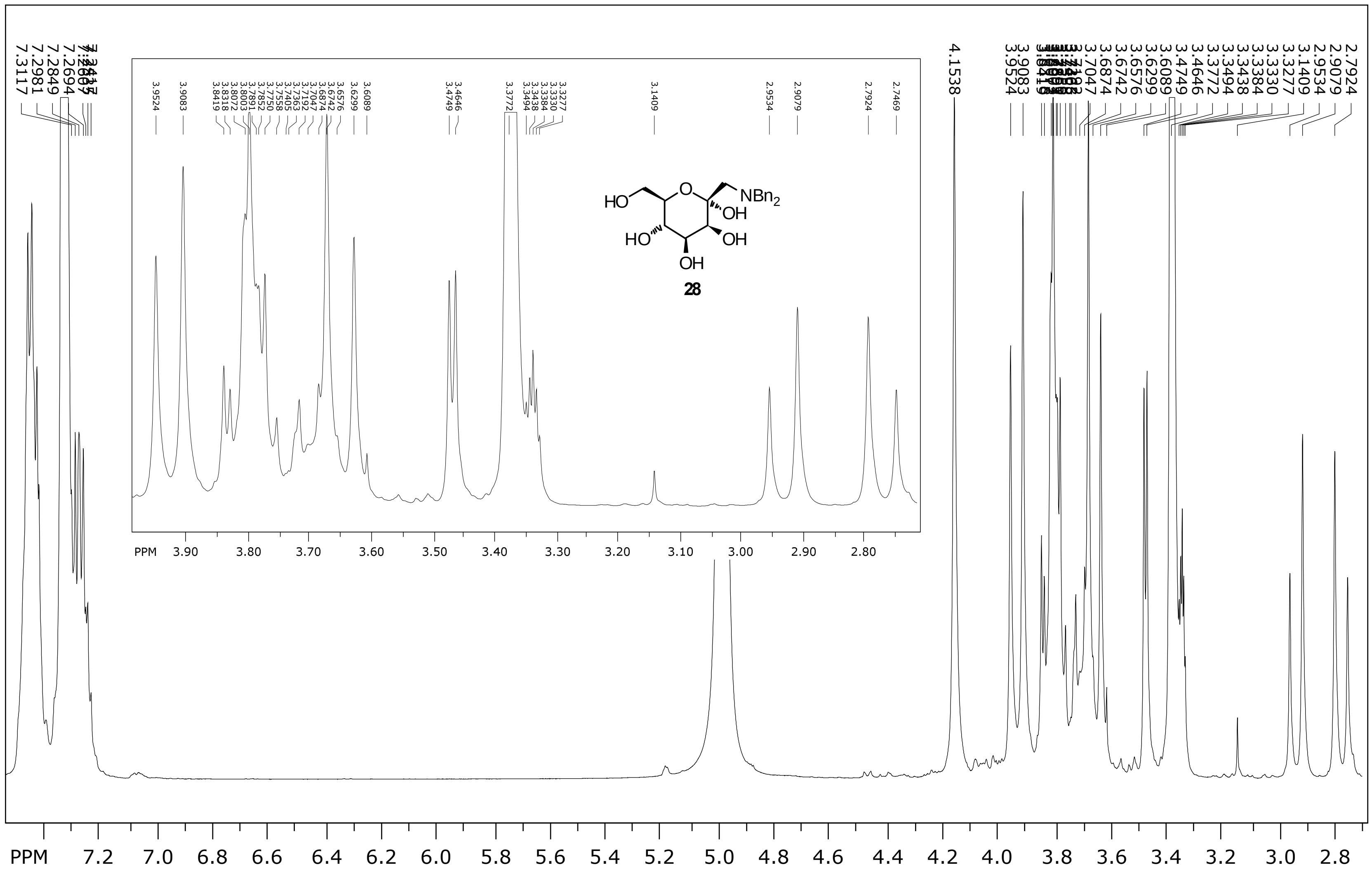

1-(*N*,*N*-Dibenzylamino)-1-deoxy-α-D-*manno*-hept-2-ulopyranose (28) ^{13}C, 75 MHz, MeOH-d_4

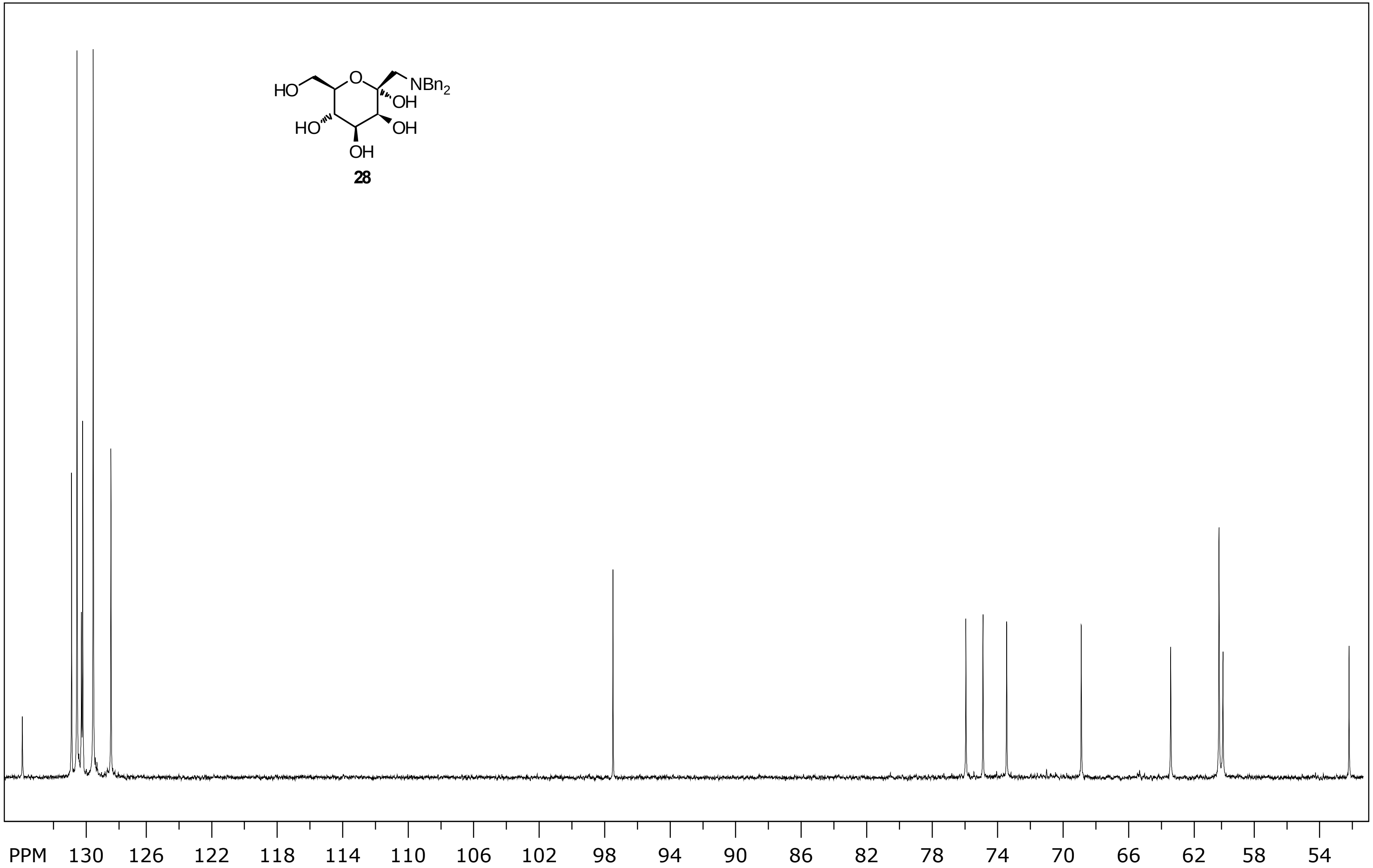

1-(*N*-(6-Hydroxyhexyl)amino)-1-deoxy-α-D-*manno*-hept-2-ulose (29) ^{1}H, 300 MHz, MeOH-d_4

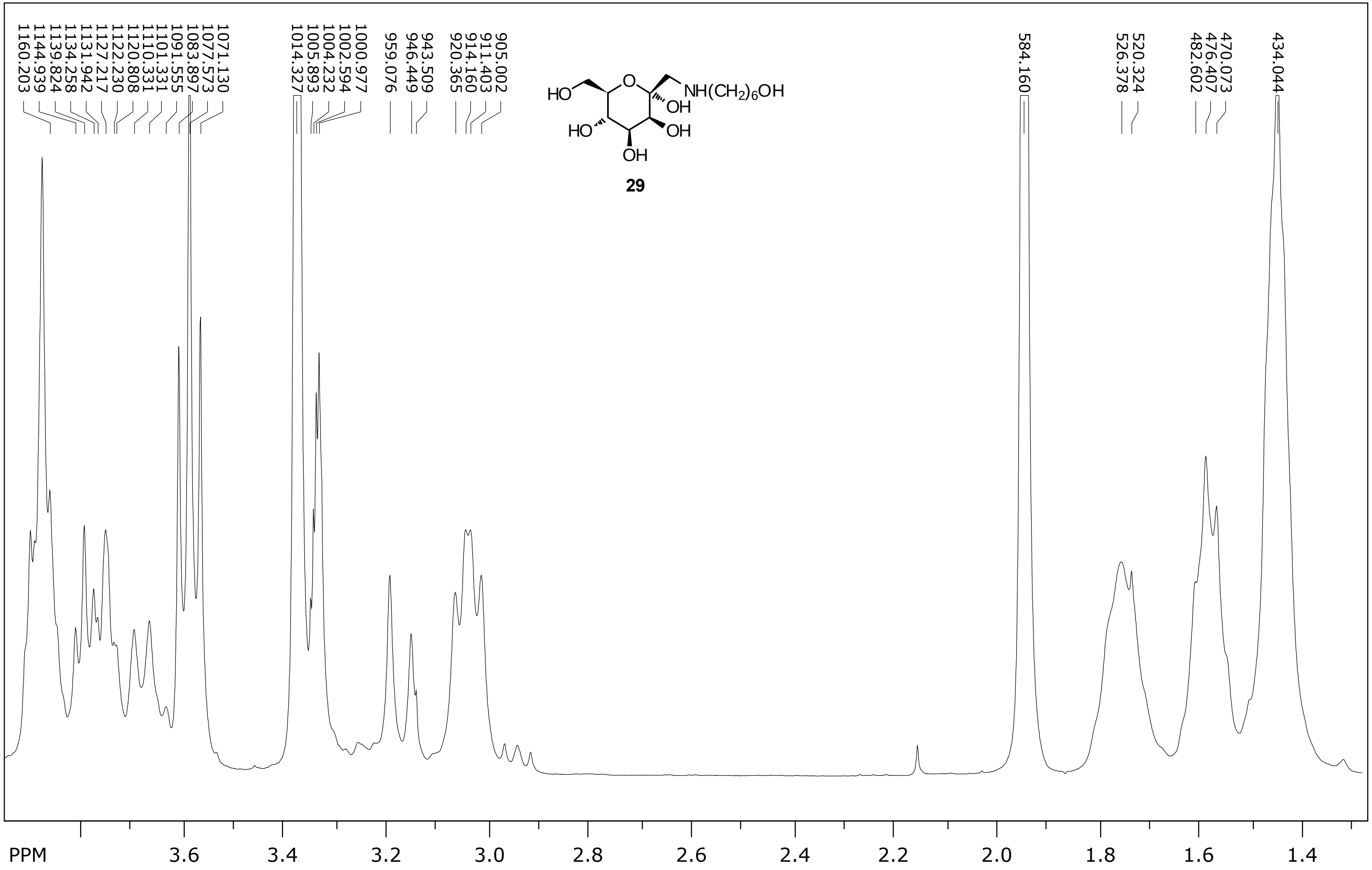

1-(*N*-(6-Hydroxyhexyl)amino)-1-deoxy-α-D-*manno*-hept-2-ulose (29) ^{13}C, 75 MHz, MeOH-d_4

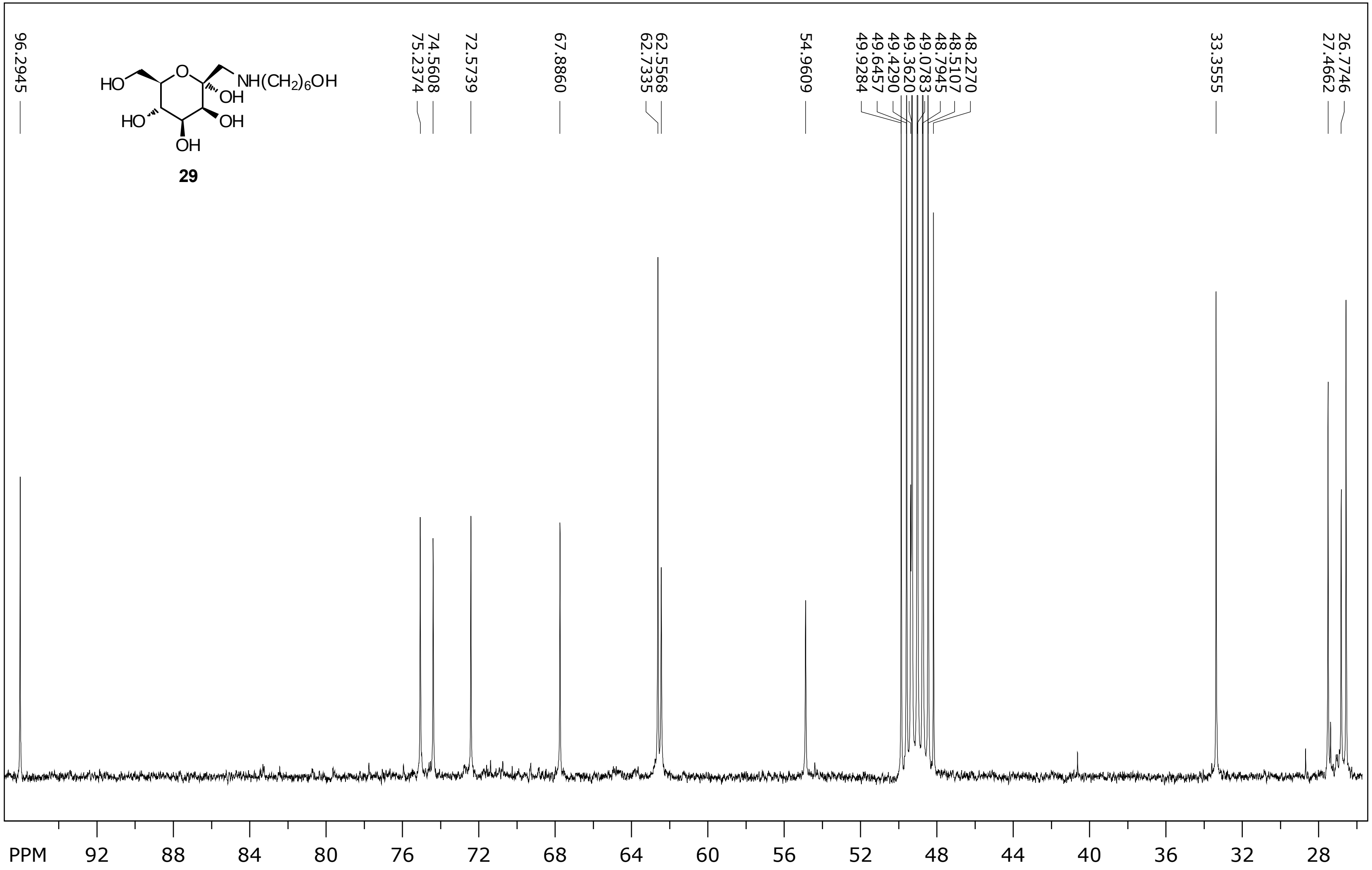

1-(*N*-(6-Hydroxyhexyl)amino)-1-*N*,2-*O*-carbonyl-1-deoxy-β-D-*manno*-pyranose (30) ^{1}H, 300 MHz, MeOH-d_4

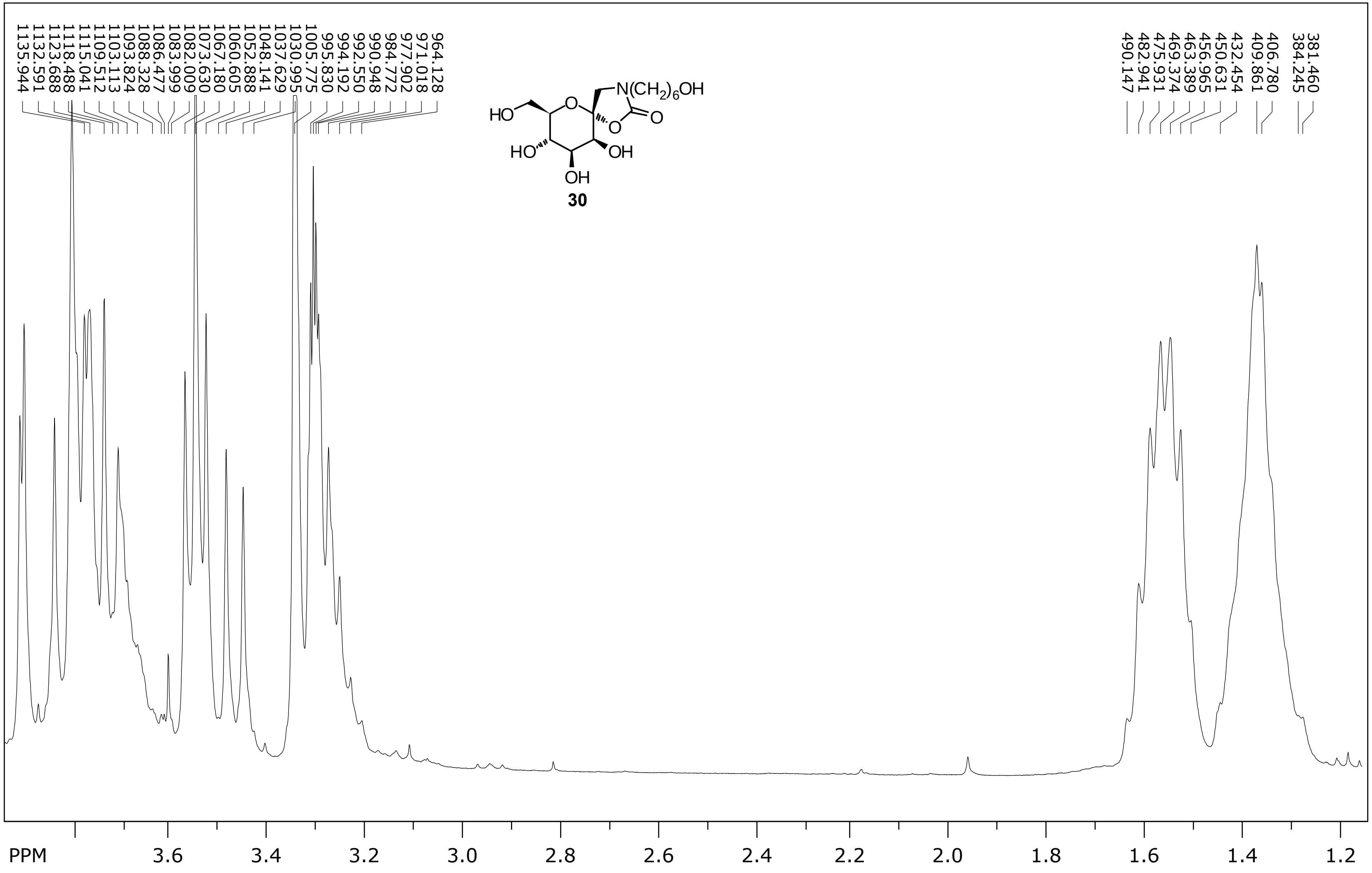

1-(*N*-(6-Hydroxyhexyl)amino)-1-*N*,2-*O*-carbonyl-1-deoxy-β-D-*manno*-pyranose (30) ^{13}C, 75 MHz, MeOH-d_4

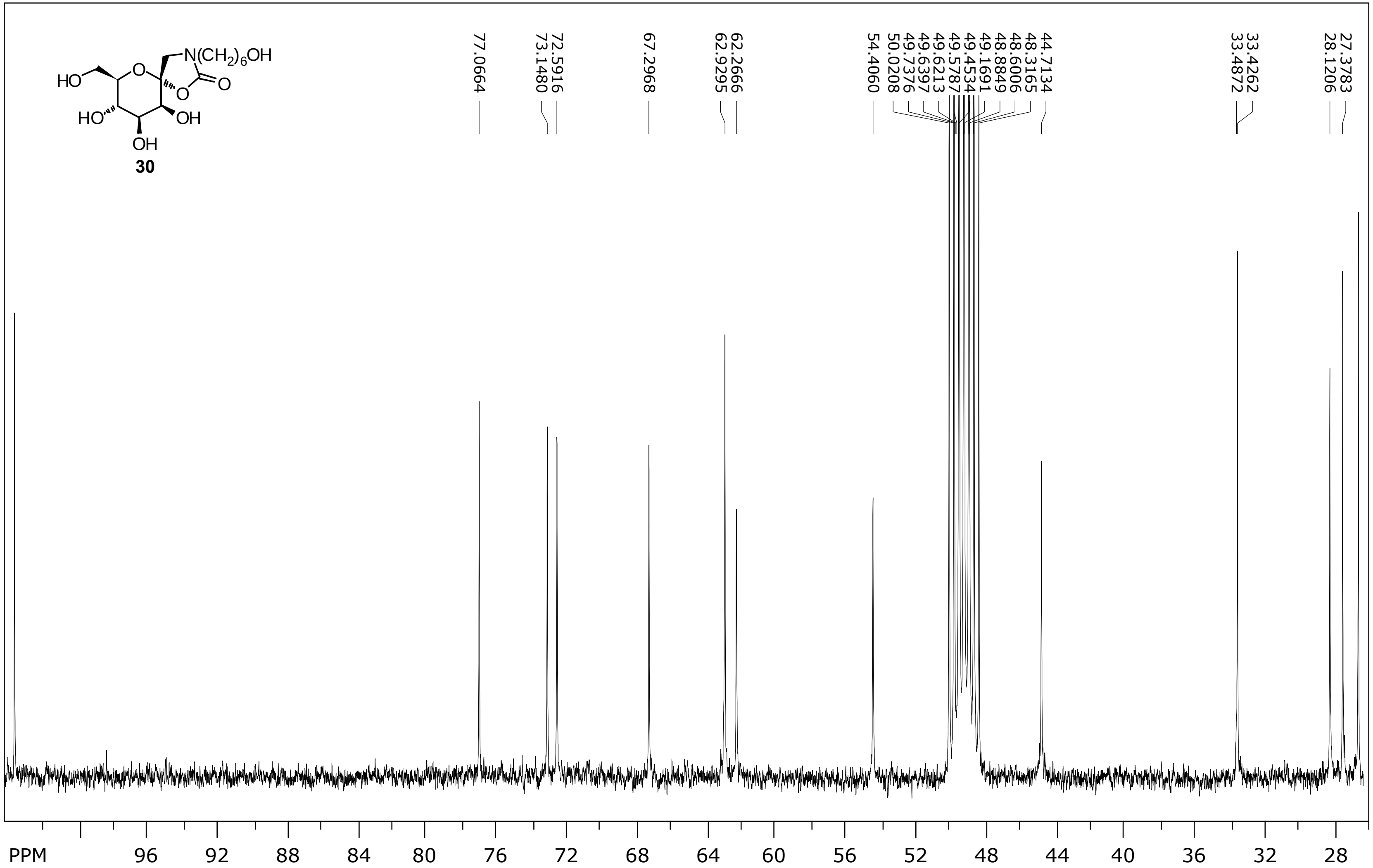

1-(*N*-(5-(Methoxycarbonyl)pentyl)amino)-1-deoxy-α-D-*manno*-hept-2-ulopyranose (31) ^{1}H, 300 MHz, MeOH-d_4

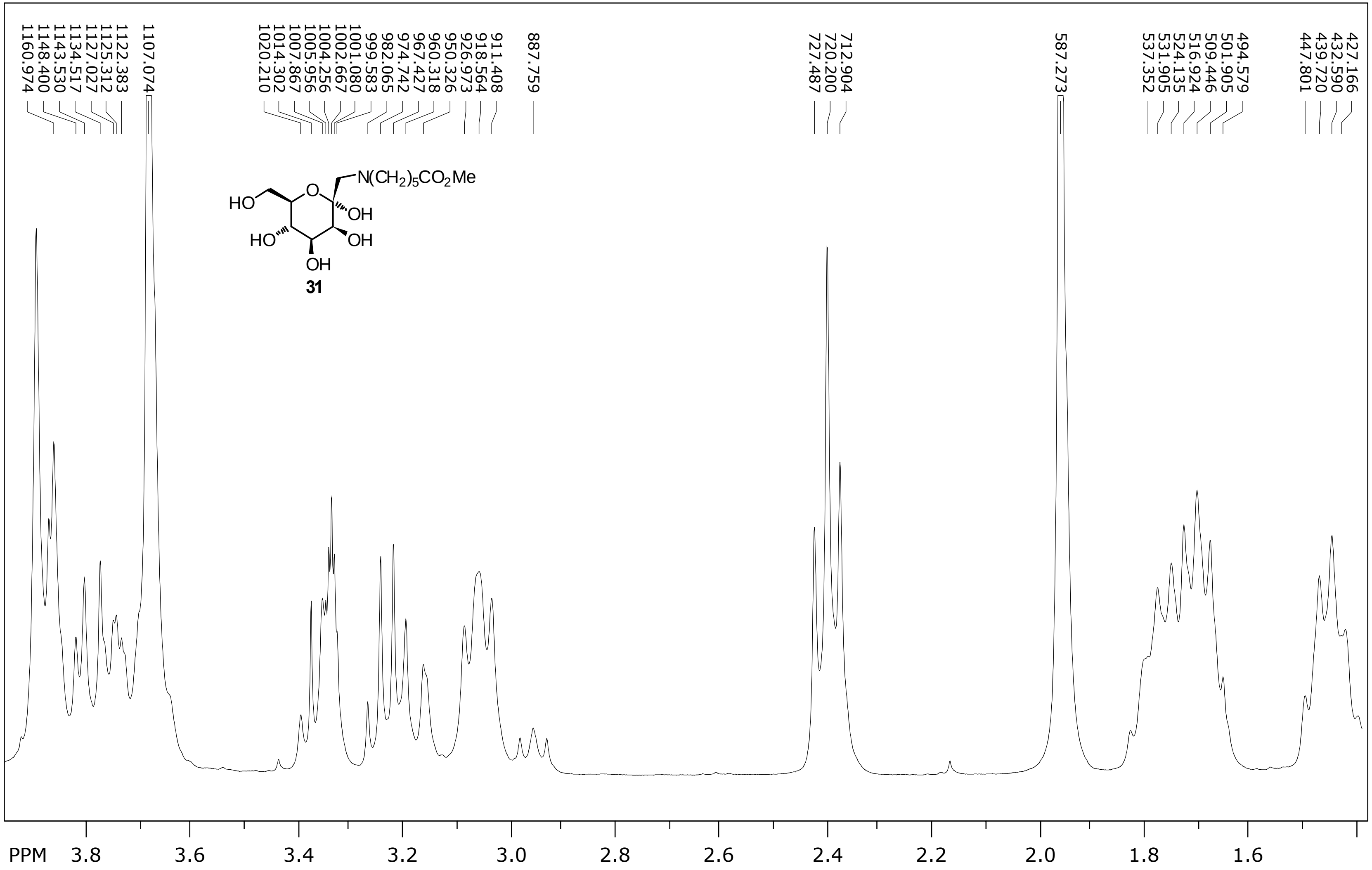

1-(*N*-(5-(Methoxycarbonyl)pentyl)amino)-1-deoxy-α-D-*manno*-hept-2-ulopyranose (31) ^{13}C, 75 MHz, MeOH-d_4

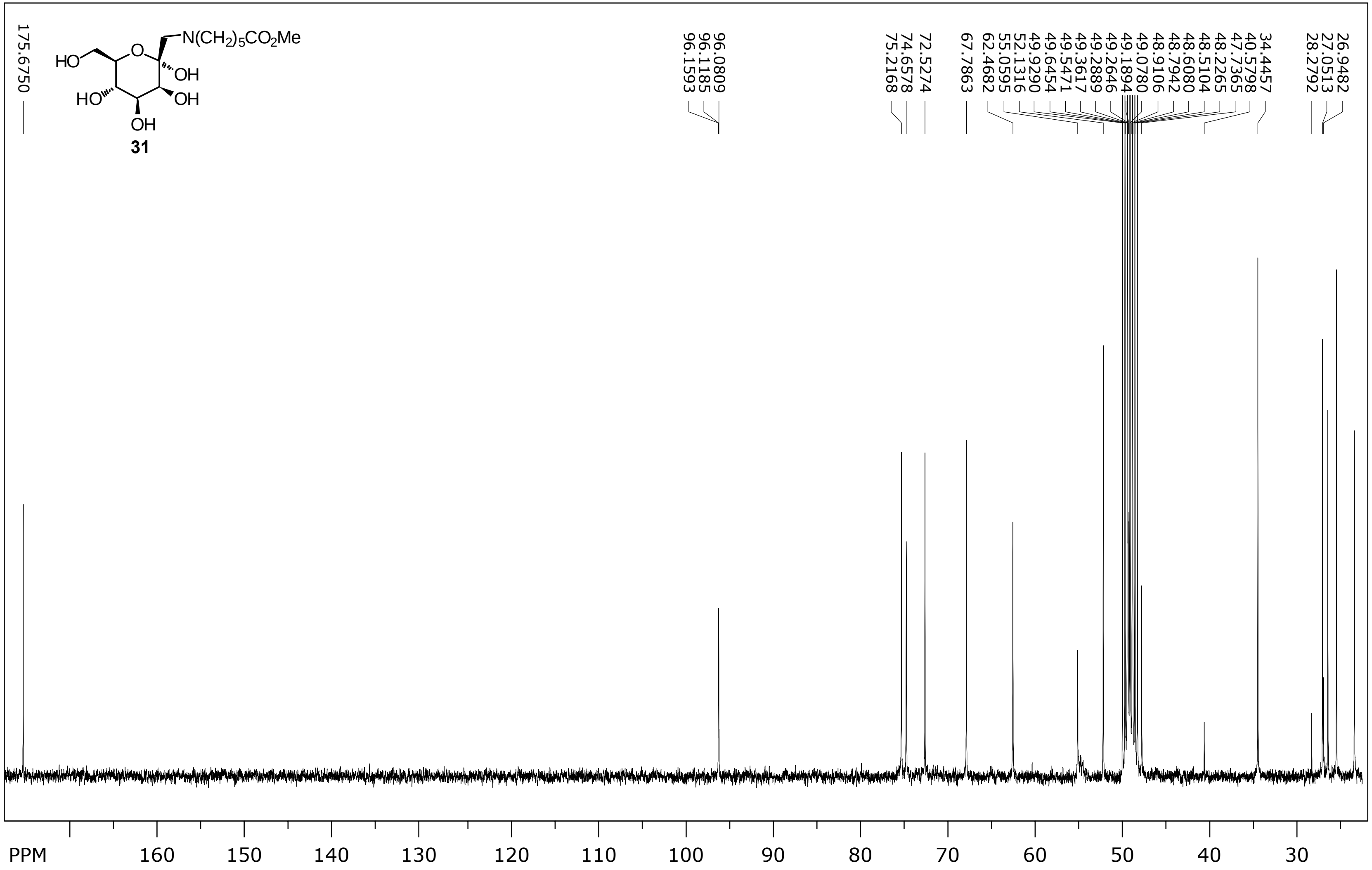

1-(*N*-(5-(Methoxycarbonyl)pentyl)amino)-1-*N*,2-*O*-carbonyl-1-deoxy-β-D-manno-hept-2-ulopyranose (32) ^{1}H, 300 MHz, MeOH-d_4

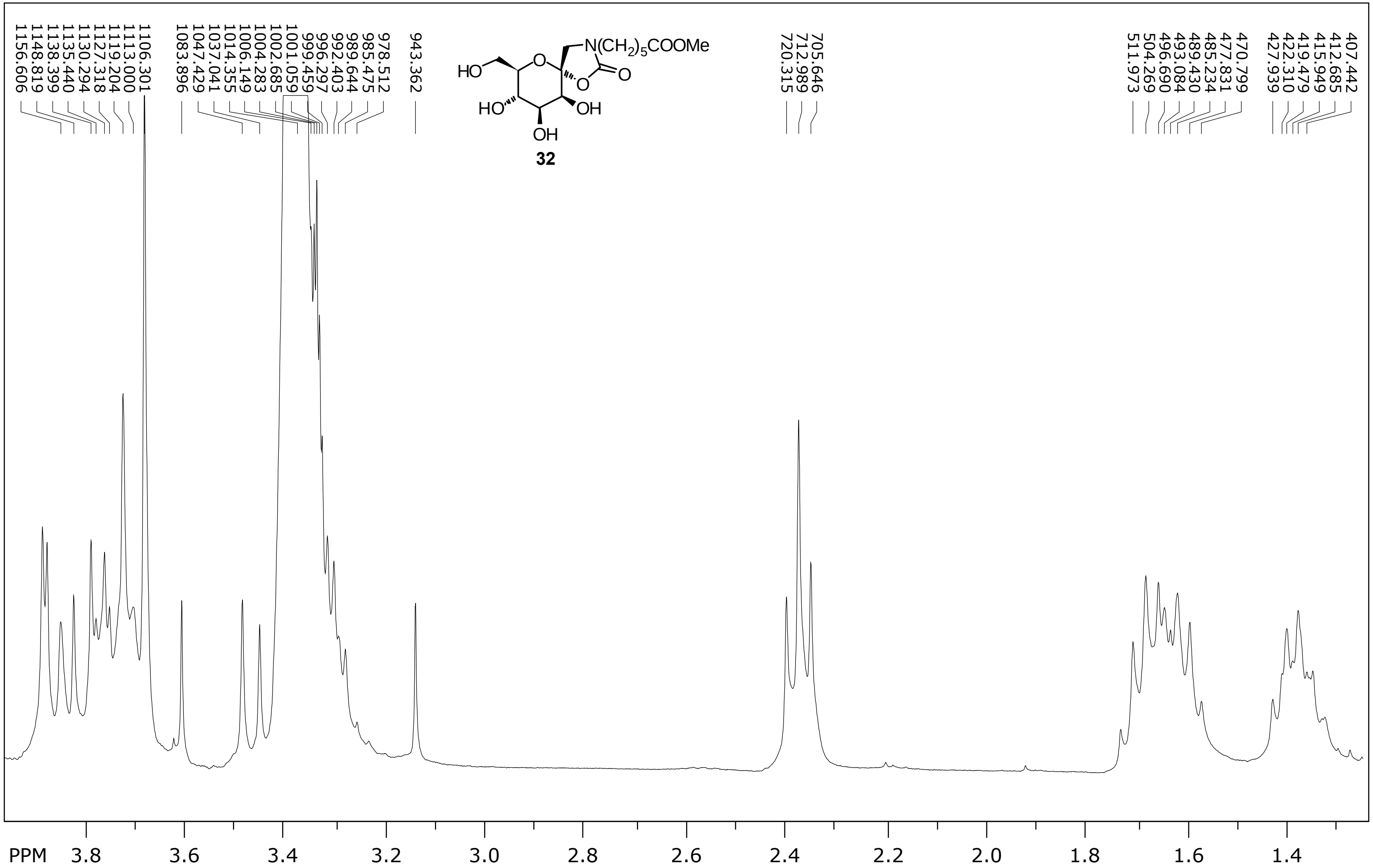

1-(*N*-(5-(Methoxycarbonyl)pentyl)amino)-1-*N*,2-*O*-carbonyl-1-deoxy-β-D-manno-hept-2-ulopyranose (32) ^{13}C, 75 MHz, MeOH-d_4

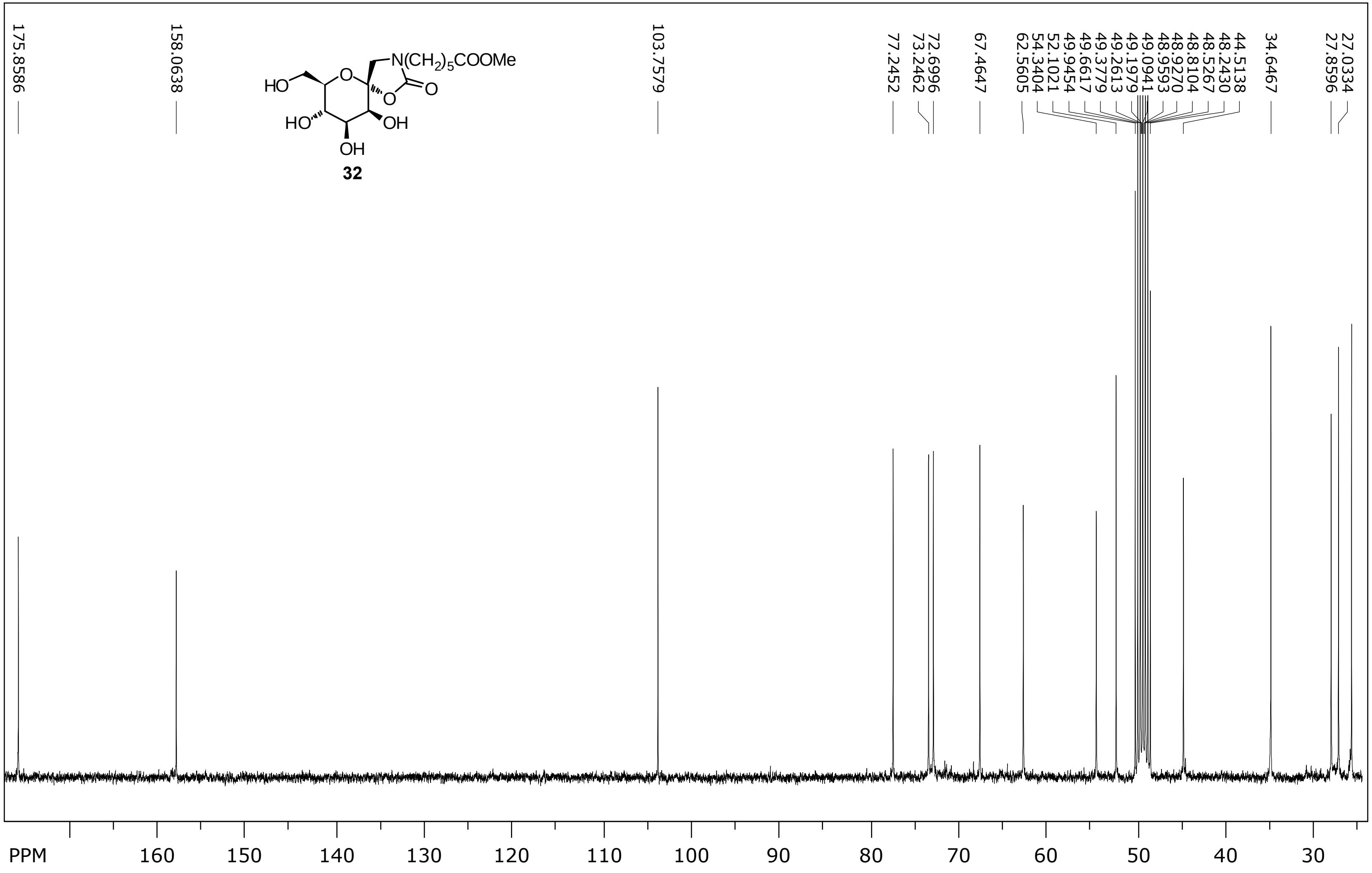

1-(*N*,*N*-Dibenzylamino)-3-acetamino-1,2-dideoxy-α,D-*gluco*-hept-2-ulopyranose (33a) and –furanose (33b) ^{1}H, 300 MHz, MeOH-d_4

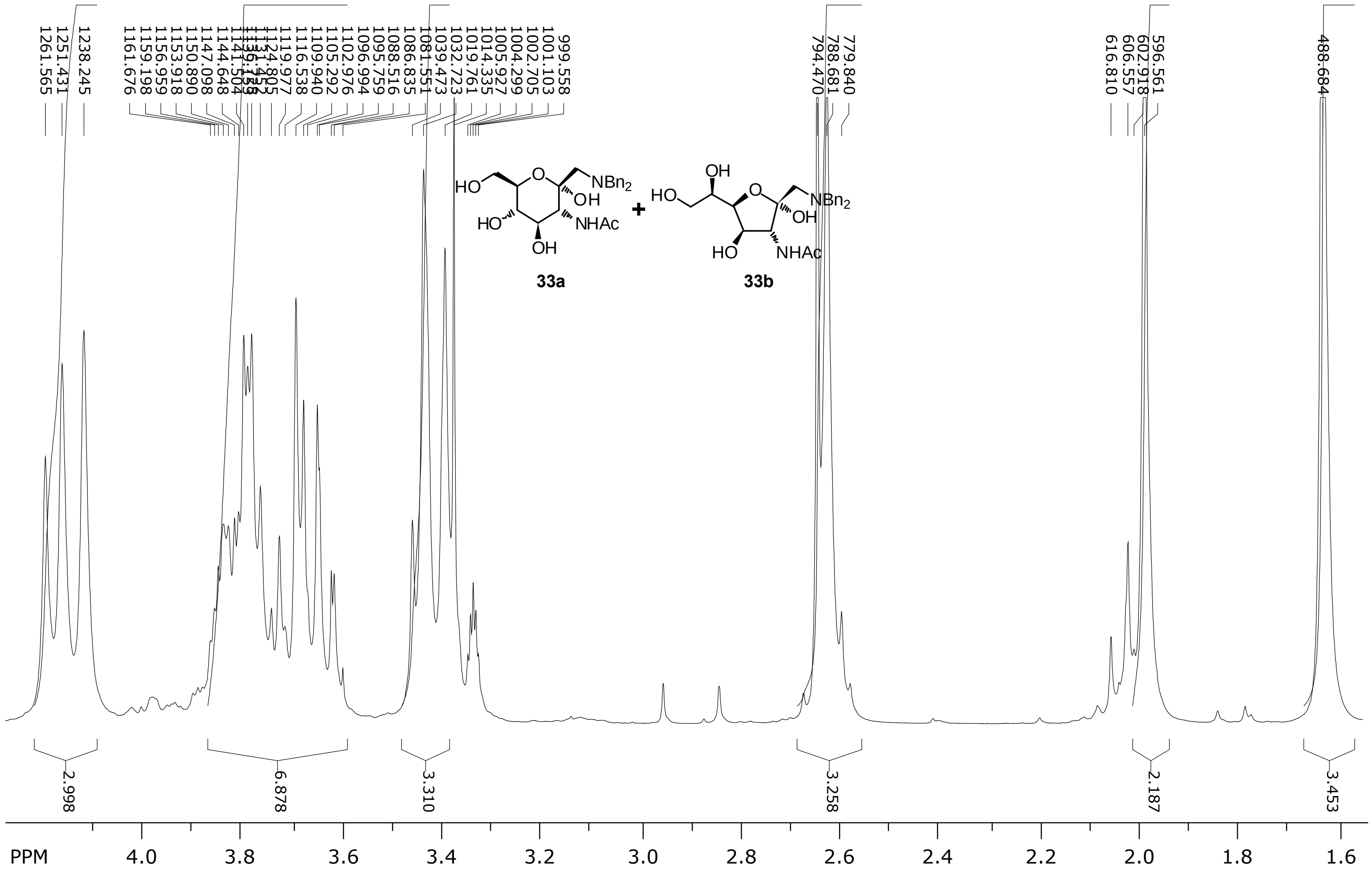

1-(*N*,*N*-Dibenzylamino)-3-acetamino-1,2-dideoxy-α,D-*gluco*-hept-2-ulopyranose (33a) and –furanose (33b) ^{13}C, 75 MHz, MeOH-d_4

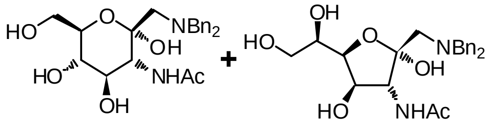

33a 33b

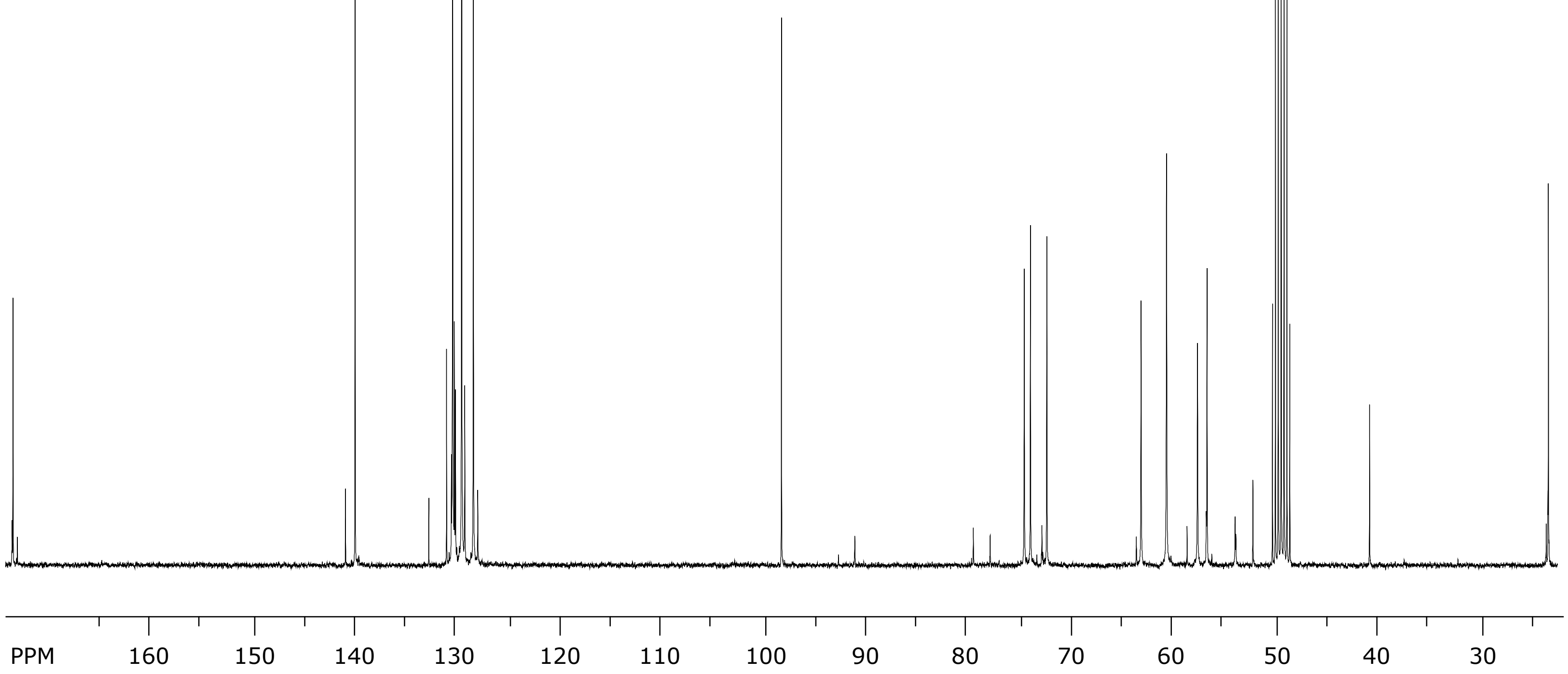

1-(*N*-(6-Hydroxyhexyl)amino)-3-acetamino-1,2-dideoxy-α-D-*gluco*-hept-2-ulopyranose (34) ^{1}H, 300 MHz, MeOH-d_4

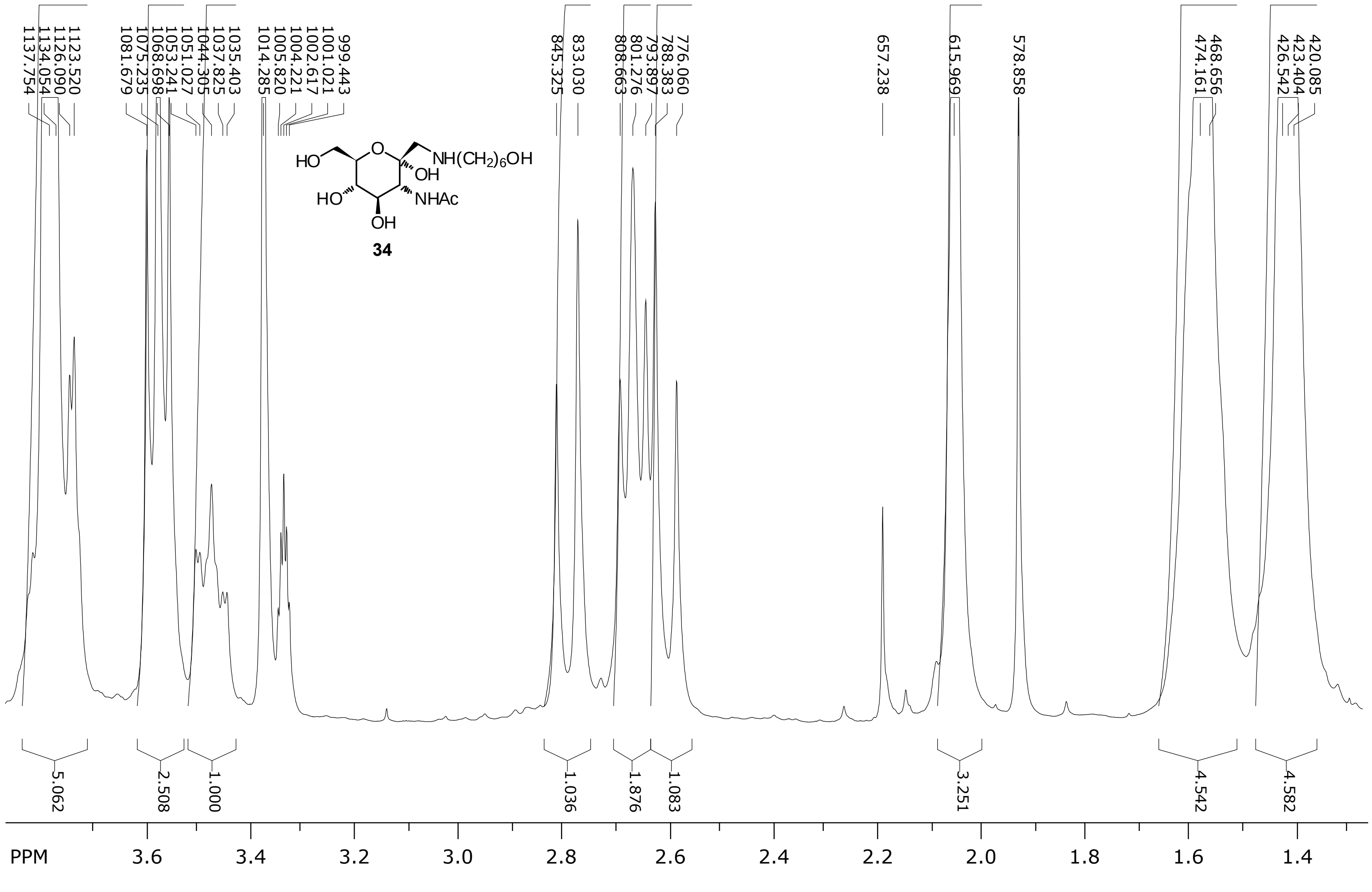

1-(*N*-(6-Hydroxyhexyl)amino)-3-acetamino-1,2-dideoxy-α-D-*gluco*-hept-2-ulopyranose (34) ^{13}C, 75 MHz, MeOH-d_4

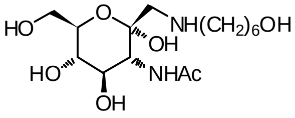

34

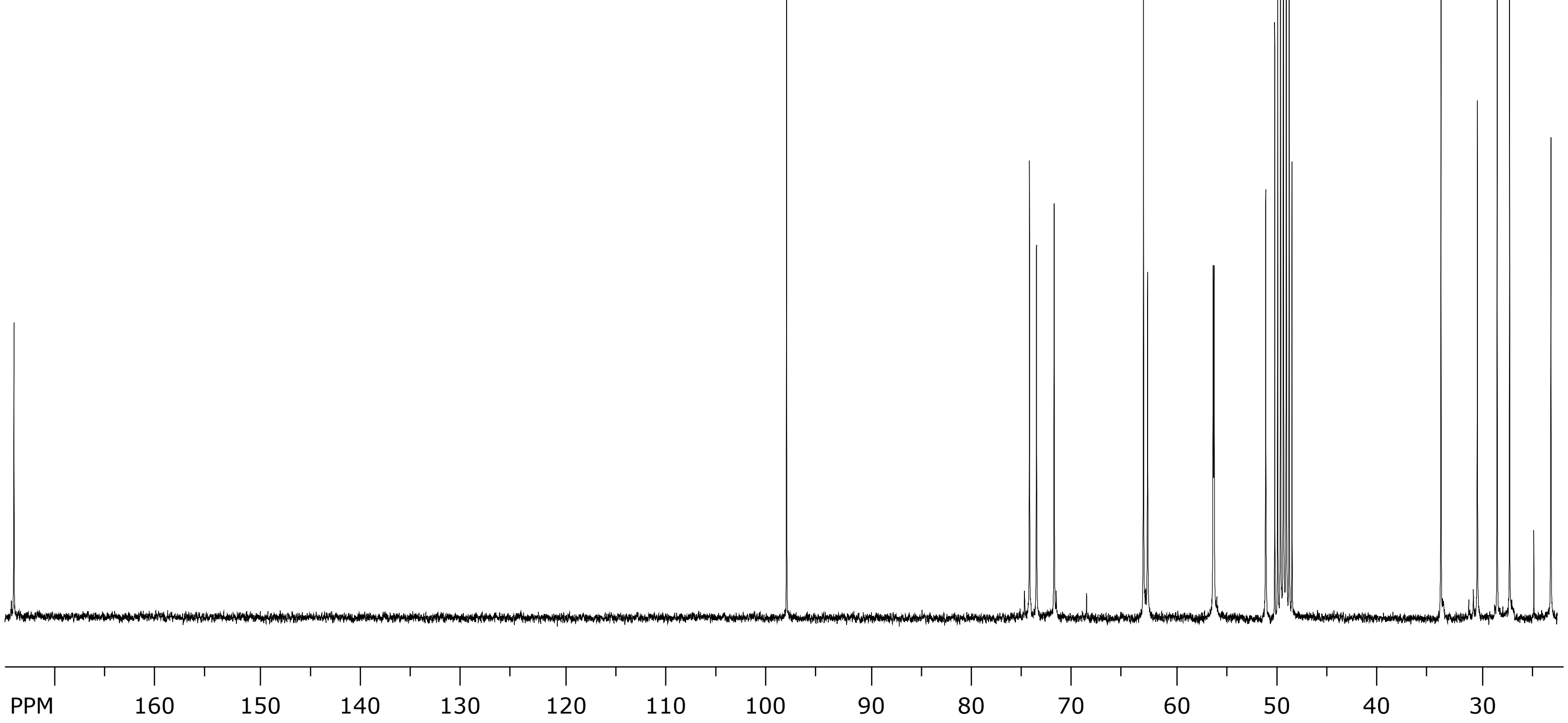

1-(*N*-(5-(Methoxycarbonyl)pentyl)amino)-2-acetamino-1,2-dideoxy-α-D-*gluco*-hept-2-ulopyranose (35) ^{1}H, 300 MHz, MeOH-d_4

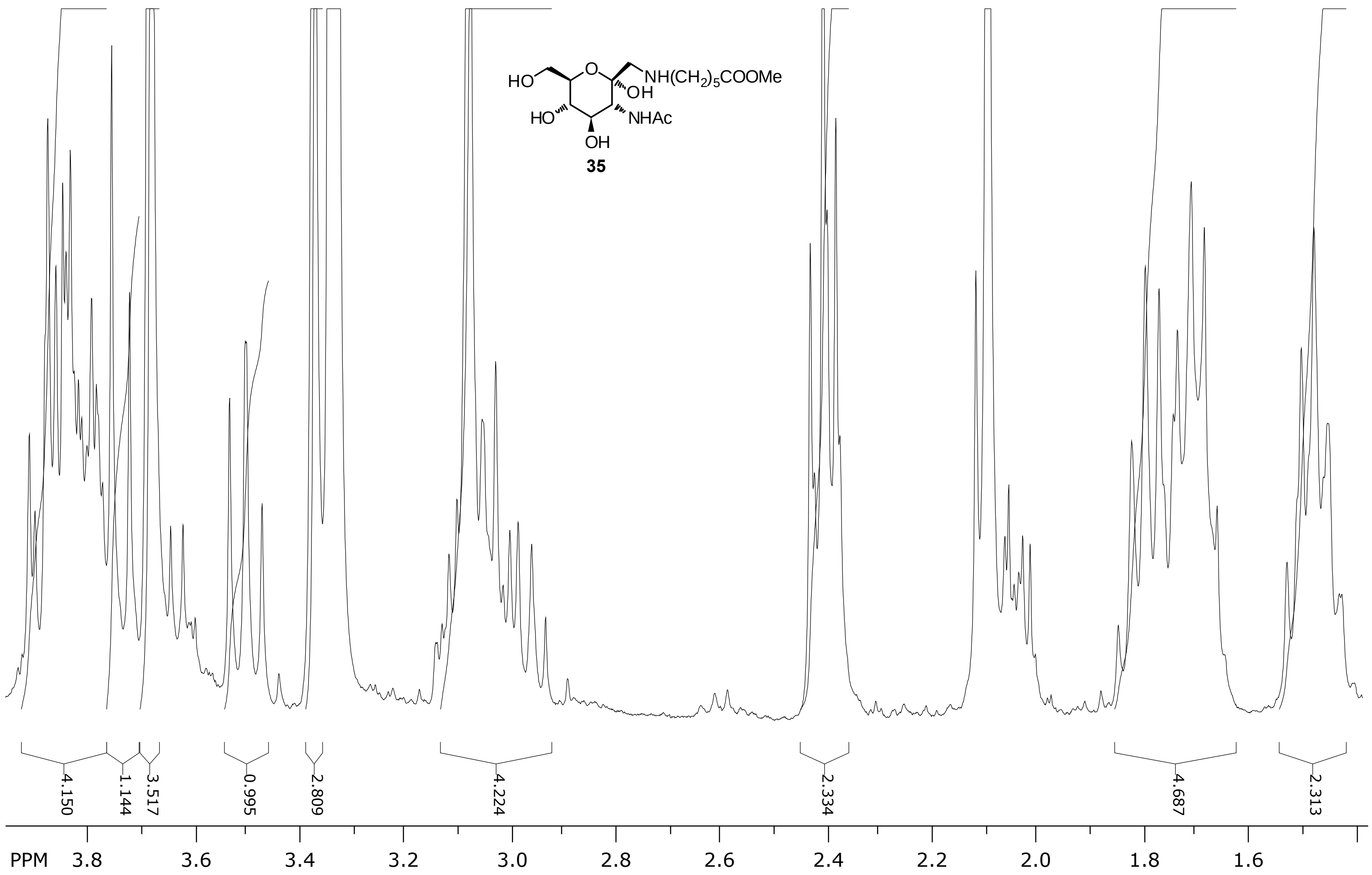

1-(*N*-(5-(Methoxycarbonyl)pentyl)amino)-2-acetamino-1,2-dideoxy-α-D-*gluco*-hept-2-ulopyranose (35) ^{13}C, 75 MHz, MeOH-d_4

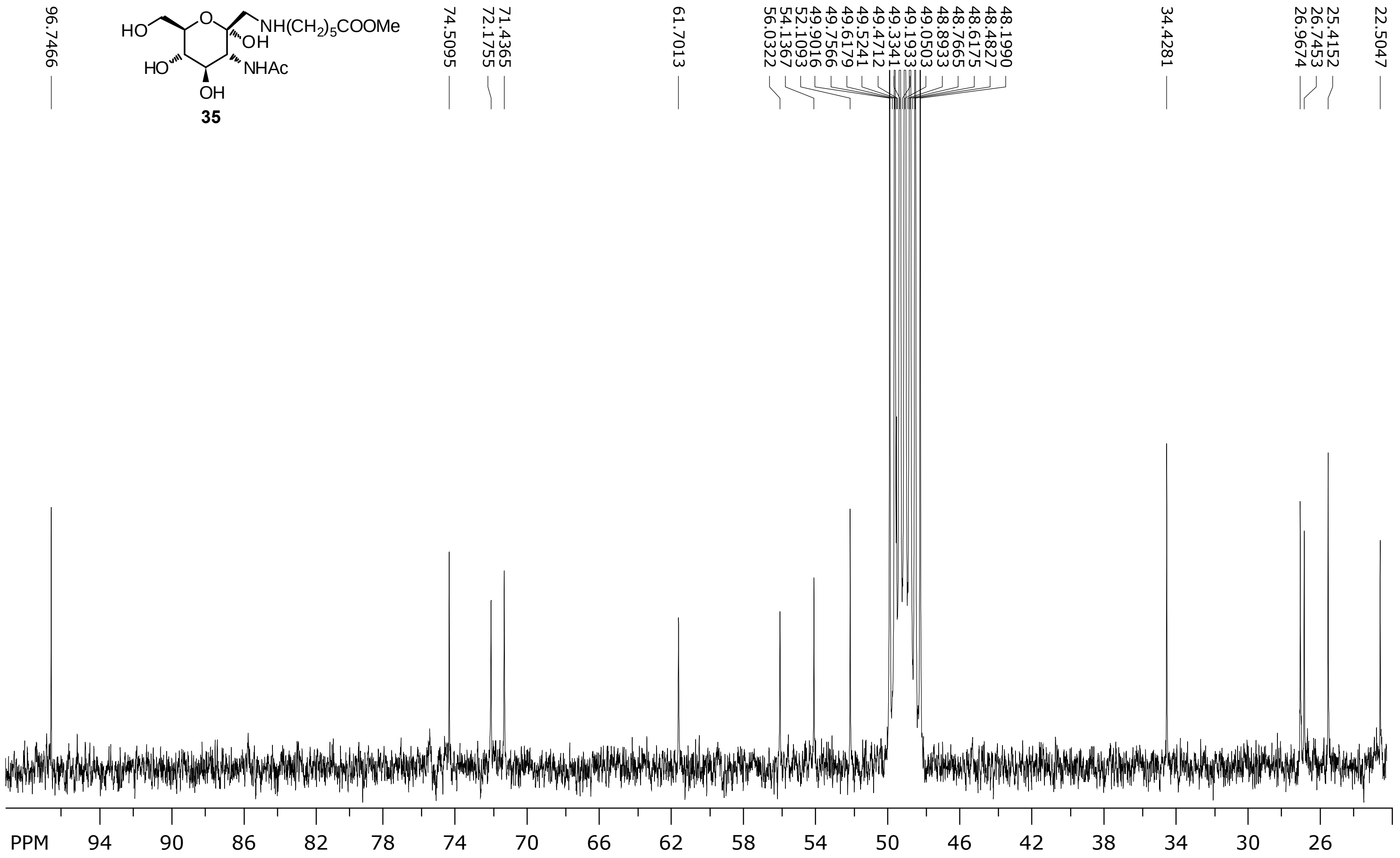

1-*N*-[(5*S*)-(*tert*-Butoxylcarbonylamino)-5-(methoxycarbonyl)pentyl]amino-3-acetamino-1,2-dideoxy-α-D-*gluco*-hept-2-ulopyranose (36)
^{1}H, 300 MHz, MeOH-d_4

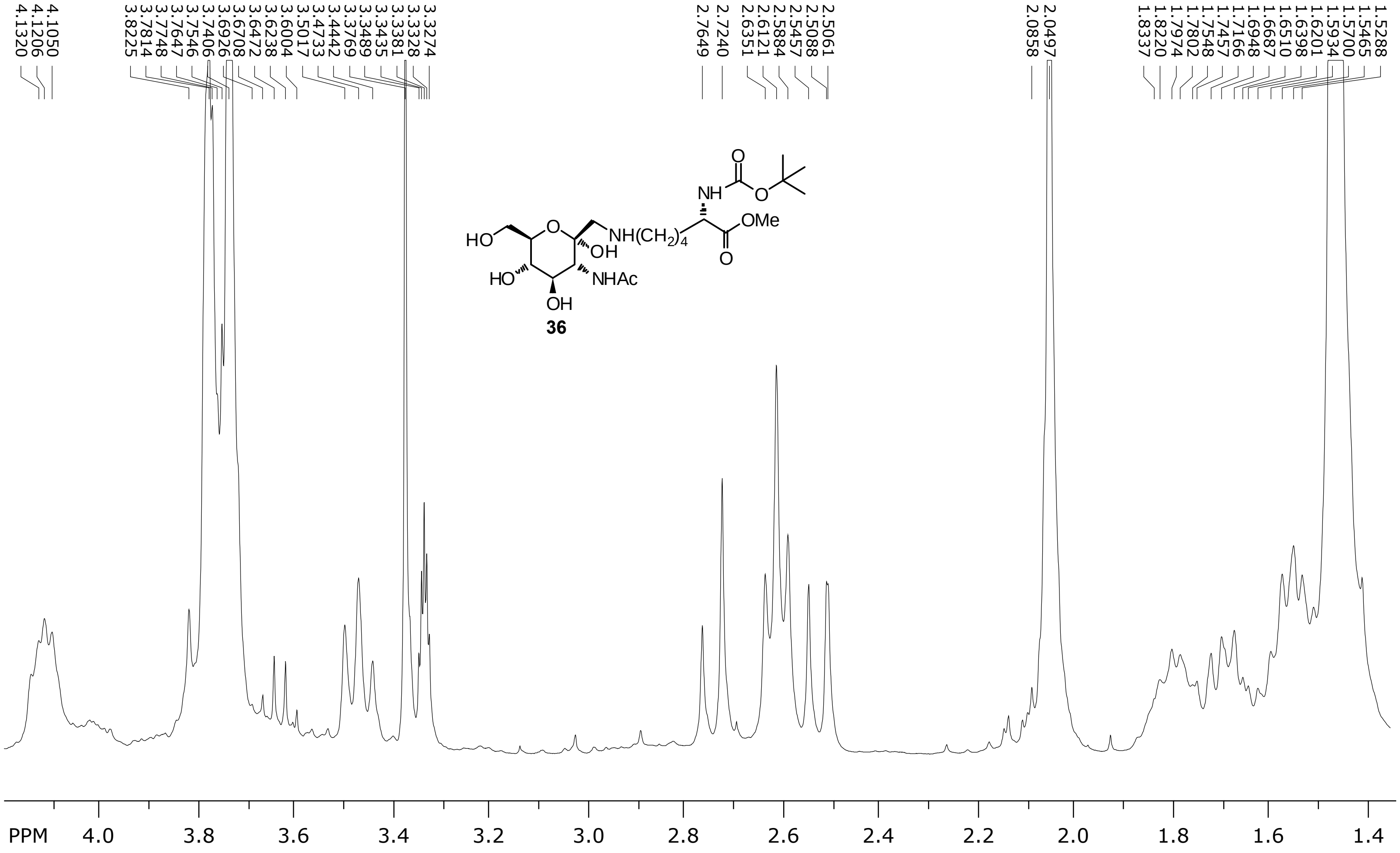

1-*N*-[(5*S*)-(*tert*-Butoxylcarbonylamino)-5-(methoxycarbonyl)pentyl]amino-3-acetamino-1,2-dideoxy-α-D-*gluco*-hept-2-ulopyranose (36)
^{13}C, 75 MHz, MeOH-d_4

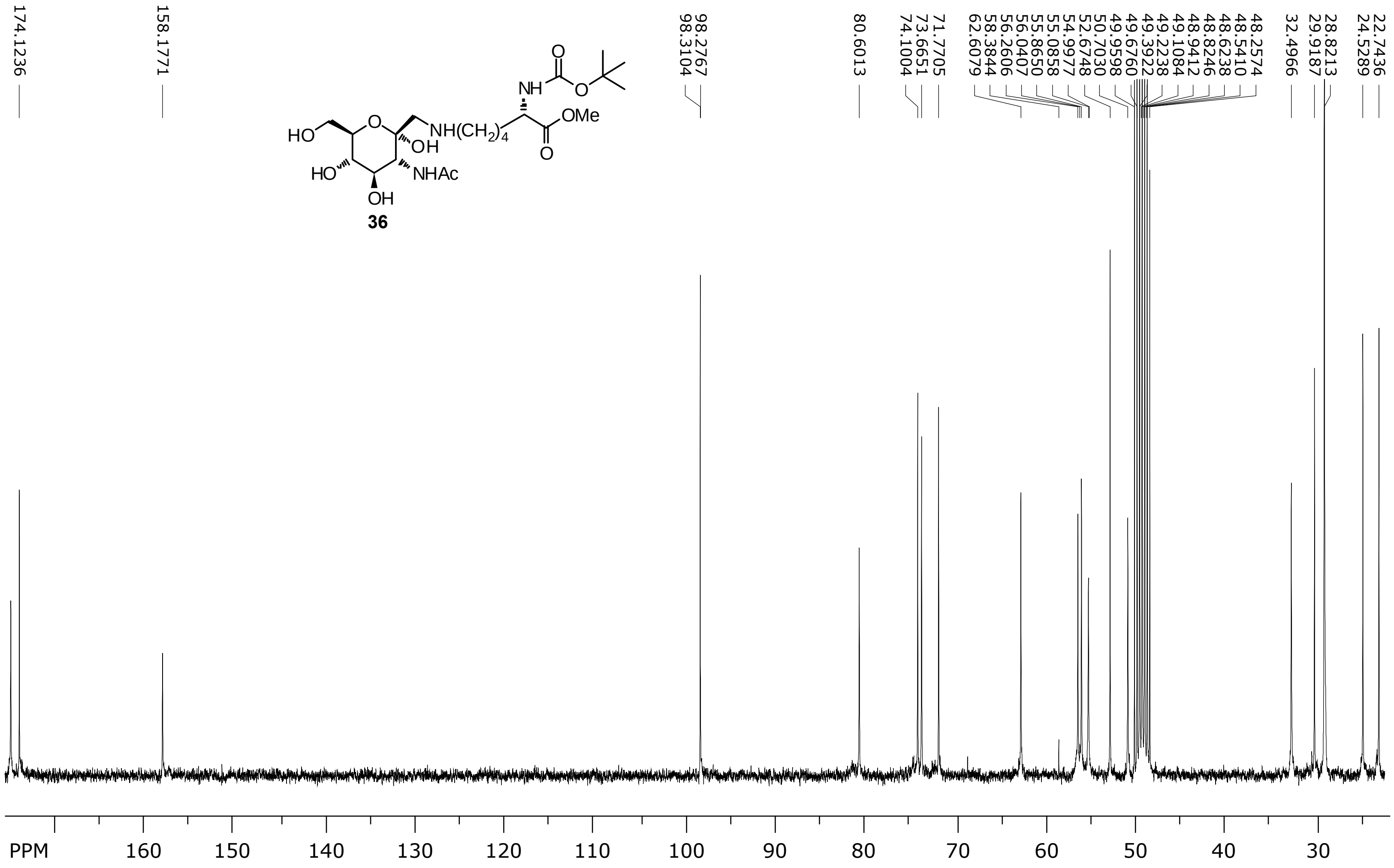

Supplement: File 1 — NMR data of compounds 16, 19, 23–36. [file Beilstein_J_Org_Chem-08-1619-s001.pdf]
